# Multi-fidelity prediction of molecular optical peaks with deep learning†

**DOI:** 10.1039/d1sc05677h

**Published:** 2022-01-04

**Authors:** Kevin P. Greenman, William H. Green, Rafael Gómez-Bombarelli

**Affiliations:** Department of Chemical Engineering, Massachusetts Institute of Technology 77 Massachusetts Ave Cambridge MA 02139 USA; Department of Materials Science and Engineering, Massachusetts Institute of Technology 77 Massachusetts Ave Cambridge MA 02139 USA rafagb@mit.edu

## Abstract

Optical properties are central to molecular design for many applications, including solar cells and biomedical imaging. A variety of *ab initio* and statistical methods have been developed for their prediction, each with a trade-off between accuracy, generality, and cost. Existing theoretical methods such as time-dependent density functional theory (TD-DFT) are generalizable across chemical space because of their robust physics-based foundations but still exhibit random and systematic errors with respect to experiment despite their high computational cost. Statistical methods can achieve high accuracy at a lower cost, but data sparsity and unoptimized molecule and solvent representations often limit their ability to generalize. Here, we utilize directed message passing neural networks (D-MPNNs) to represent both dye molecules and solvents for predictions of molecular absorption peaks in solution. Additionally, we demonstrate a multi-fidelity approach based on an auxiliary model trained on over 28 000 TD-DFT calculations that further improves accuracy and generalizability, as shown through rigorous splitting strategies. Combining several openly-available experimental datasets, we benchmark these methods against a state-of-the-art regression tree algorithm and compare the D-MPNN solvent representation to several alternatives. Finally, we explore the interpretability of the learned representations using dimensionality reduction and evaluate the use of ensemble variance as an estimator of the epistemic uncertainty in our predictions of molecular peak absorption in solution. The prediction methods proposed herein can be integrated with active learning, generative modeling, and experimental workflows to enable the more rapid design of molecules with targeted optical properties.

## Abstract 2

A multi-fidelity deep learning approach that utilizes data from both experiments and physics-based calculations predicts molecular absorption peaks with higher accuracy and generalizability than existing methods.

## Graphical abstract

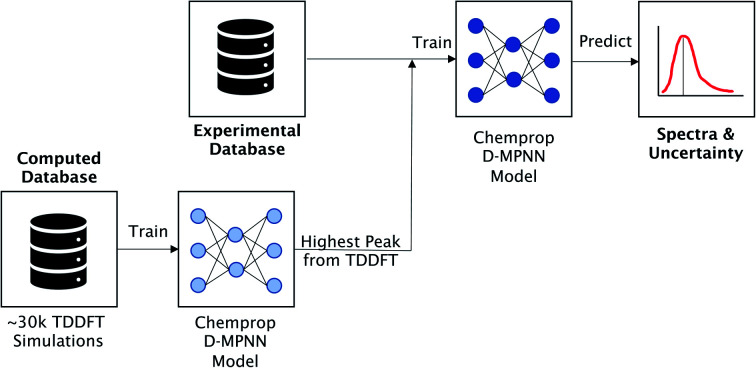

## 1 Introduction

Dye molecules are used in many applications ranging from sensitizers for solar cells to biomedical imaging and diagnostics.^1,2^ The optical properties of dyes, namely their absorption and emission characteristics, must be known to determine their suitability for particular applications. Although numerous theoretical and statistical methods exist to predict these properties, many of these methods are not sufficiently accurate or general, or require significant computational cost, all of which hinder their application to large and diverse sets of molecules. Herein, we propose new deep learning methods that use learned dye and solvent representations and multi-fidelity data to improve prediction accuracy and generalizability on rigorous splits of several of the largest open-source datasets. Our models are publicly available for making predictions with corresponding uncertainty estimates.

Many theoretical methods have been developed for predicting molecular optical properties, including empirical tables, semi-empirical methods, time-dependent density functional theory (TD-DFT), and wavefunction-based methods.^3,4^ TD-DFT has been the most widely used method for at least the past decade because of its favorable accuracy/cost trade-off and its capacity to be coupled with continuum solvents approximations,^5^ and it has been benchmarked and reviewed extensively.^6,7^ In parallel to theoretical methods, researchers have also developed surrogate statistical models that predict UV/Vis spectra from molecular structure at a lower computational cost than TD-DFT. ML studies for predicting properties related to the electronically excited states of molecules have been reviewed recently.^8,9^

Limitations in previous statistical modeling efforts can be classified into three categories: data sparsity, molecular representations, and solvent representations.^10^ Many studies have focused on a narrow part of chemical space (*e.g.* a single dye family) because of the limited availability of large UV/Vis datasets. This data sparsity has been addressed recently with the publication of several experimental datasets,^1,11–18^ described in Table 1. There are also several large computed datasets of excitation energies available (Table 2). However, studies are still lacking on how the chemical diversity of the training data impacts model performance on new, unrelated chemical space. Many prediction methods have created molecular representations based on generic structure-based fingerprints or human-selected descriptor features. Most previous studies did not consider solvent effects, but many leveraged descriptors derived from quantum chemical calculations. The issue of data sparsity is related to the shortcomings of solvent representations in previous models; with relatively few examples of dyes measured in more than one solvent, it was sometimes easier to train a model only on data in the most commonly reported solvent to remove this complexity from the model.

**Table 1:** Existing datasets of experimental UV/Vis spectroscopic properties. The properties listed for each dataset are not necessarily present for every measurement. “Full” refers to the full absorption/emission spectrum as *xy*-coordinate pairs, *λ*_max_ is the peak wavelength, *ε*_max_ is the peak molar attenuation coefficient (also called the molar extinction coefficient or molar absorptivity), *σ* is the peak FWHM (bandwidth), *Φ* is the quantum yield, and *τ* is the fluorescence lifetime. A subset of the data in the ChemFluor^16^ set was extracted from the Fluorophores^12^ set. The number of entries for the UV/Vis+ dataset includes the count of the dye entries only, and the entries for NIST do not include ions

Dataset	Entries	Dye	Solvent	Absorption	Emission	Other
ChemDataExtractor^14^	8467	SMILES	Name	*λ*_max_, *ε*_max_	—	—
ChemFluor^16^	4386	SMILES	Name	*λ*_max_	*λ*_max_	*Φ*
Deep4Chem^17^	20 236	SMILES	SMILES	*λ*_max_, *σ*_abs_, *ε*_max_	*λ*_max_, *σ*_emi_	*Φ*, *τ*
DSSCDB^1^	5178	SMILES	Name	*λ*_max_	*λ*_max_	—
Dye aggregation^15^	4043	SMILES	Name	*λ*_max_	—	—
Fluorophores.org^12^	955	Name	Name	Full, *λ*_max_	Full, *λ*_max_	*Φ*, *τ*
NIST^11^	2306	MOL file	—	Full	—	—
PhotochemCAD^13^	552	Name	Name	*λ*_max_, *ε*_max_	—	*Φ*
UV/Vis+^18^	112	Name	Name	Full	Full	—

**Table 2:** Existing large datasets of computed excitation energies. Each dataset contains additional properties beyond excitation energies, such as oscillator strengths, highest occupied molecular orbital (HOMO), and lowest unoccupied molecular orbital (LUMO), but the set of reported properties is different for each dataset. Some datasets report results from multiple levels of theory (ranging from semi-empirical to coupled cluster), but all were calculated in vacuum. Many smaller datasets and datasets containing only ground-state properties (*e.g.* HOMO and LUMO) exist that are not referenced here

Dataset	Entries	Molecule
QM7b^19,20^	7211	Coulomb matrix
QM8 (ref. 21 and 22)	21 786	XYZ file
QM-symex^23^	172 736	XYZ file
PubChemQC^24^	3 411 649	MOL file

Among the previous studies on predicting absorption peak wavelengths or excitation energies, the work of Ju *et al.*,^16^ Kang *et al.*,^25^ and Joung *et al.*^26^ is particularly noteworthy because of the size of their training datasets and the accuracies this enabled them to achieve. Ju *et al.* trained a gradient boosted regression tree (GBRT) algorithm on composite fingerprints to predict the maximum absorption and emission wavelengths and photoluminescence quantum yield (PLQY) using a large set of experimental data they compiled from the literature. Kang *et al.* trained a random forest algorithm on a subset of the PubChemQC database^24^ to predict B3LYP/6-31G* excitation energies and oscillator strengths in a vacuum from molecular fingerprints. Joung *et al.* used their previously compiled experimental dataset^17^ to train a model that uses graph-convolutional networks (GCN) to predict multiple molecular optical properties, including the absorption and emission peak wavelengths.

Although these recent works achieved impressive accuracies, their reported performance may be more representative of how they would perform in substituent-selection applications as opposed to *de novo* design tasks with unseen chemistries. Recent reviews of ML best practices in chemistry and materials science have warned against data leakage from the same compound or composition being present under multiple measurement conditions.^27,28^ Random splitting into training/test or training/validation/test sets based on dye–solvent pair may not be sufficient for assessing model generalizability on this task because test error may be spuriously low if a dye appears in both training and test sets in different solvents or even if the molecules in the test set are too chemically similar to the training data. We set out to explore how these decisions impact model performance.

Additionally, previous work has not attempted to leverage a combination of computed and experimental data in predictions of optical spectra. This multi-fidelity approach is desirable because of the lower cost and greater availability of calculations with respect to experiments. Multi-fidelity methods have been demonstrated in several other applications for integrating data from multiple levels of theory or from theory and experiments.^29–31^ Furthermore, theoretical methods do not have a domain of applicability constrained by a training set, so they are more reliable for making predictions on chemistries with low similarities to existing data. These factors suggest that a multi-fidelity approach may improve model accuracy and generalizability on experimental predictions and may be more useful in active learning.

In this work, we leverage recently-compiled experimental datasets and directed message passing neural networks (D-MPNN)^32^ to address previous limitations with molecular and solvent representations. The D-MPNN approach learns representations for the dye and solvent that are optimized for predicting absorption properties. We compare our optimized representations to a state-of-the-art fingerprint-based method and to alternative solvent descriptors. Our method produces interpretable representations and estimates the uncertainty in its predictions. We emphasize the importance of using rigorous splitting techniques for assessing the ability of a model to generalize to unseen chemistries, and we show that incorporating results from physics-based calculations into model training improves performance across several large datasets. The predictive capability of our techniques will enable more rapid design of molecules with target optical properties.

## 2 Methods

### 2.1 Data sources and preprocessing

We compiled experimental UV/Vis absorption data from several of the largest openly-available datasets: Deep4Chem,^17^ ChemFluor,^16^ Dye Aggregation (DyeAgg),^15^ ChemDataExtractor (CDEx),^14^ and the Dye-Sensitized Solar Cell Database (DSSCDB).^1^ Among the datasets listed in Table 1, these were the largest and most easily machine-readable, and they included solvent information for each measurement. All of these data sources reported the dyes in the form of SMILES,^33^ but only the Deep4Chem set reported the solvents in this form. For the other four sets, we converted the solvent names and abbreviations to SMILES through a manually-constructed dictionary mapping because automatic tools did not recognize the necessary variety of names and abbreviations for many solvents. We extracted all measurements that included a valid dye SMILES string, solvent SMILES string, and peak wavelength of maximum absorption. We determined the validity of the SMILES strings using RDKit^34^ and dropped measurements with invalid dye or solvent SMILES (105 measurements) and those containing “.” to represent clusters of molecules (373 measurements). The remaining dataset contained 28 734 measurements. We removed any dye–solvent pairs with duplicate measurements within the same dataset that disagreed by more than 5 nm. For those that agreed within 5 nm, the mean of the values was used. This resulted in datasets of size 1825 (CDEx), 3840 (ChemFluor), 14 771 (Deep4Chem), 1720 (DSSCDB), 3025 (DyeAgg), and 24 580 (a combined set of ChemFluor, Deep4Chem, DSSCDB, and DyeAgg).

### 2.2 TD-DFT calculations

For each dye molecule in the combined experimental dataset, as well as a set of molecules with dye-like substructures parsed from USPTO patents and commercial vendors, initial geometries were generated using RDKit to convert the SMILES strings into Cartesian coordinates.^35^ These geometries were refined using semi-empirical tight-binding density functional theory (GFN2-xTB)^36^ in the ORCA software,^37^ followed by geometry optimizations at the BP86 (ref. 38)-D3 (ref. 39)/def2-SVP^40^ level of theory. Finally, TD-DFT calculations were performed with the Tamm–Dancoff approximation (TDA)^41^ at the *ω*B97X-D3 (ref. 42)/def2-SVPD level of theory in Orca. This pipeline was completed for 28 772 molecules, of which 10 409 had a corresponding experimental measurement in at least one solvent from one of the aforementioned datasets. The total number of experiments with a corresponding TD-DFT calculation in vacuum was 19 409 (including measurements of the same molecule taken in more than one solvent).

For a subset of the complete dataset (only ChemFluor, DyeAgg, CDEx, and DSSCDB), we began with the optimized geometry calculated with BP86-D3/def2-SVP in ORCA and performed an additional TD-DFT calculation at the *ω*B97XD/def2-SVP level with solvent corrections in Gaussian.^43^ The solvent calculations were done using the integral equation formalism polarizable continuum model (IEFPCM) and Gaussian defaults for excited state solvation. This pipeline was completed for 6707 dye–solvent pairs.

We extracted the peak vertical excitation energy from each of these calculations according to the following procedure: (1) if none of the energies were in the range of 1–4 eV, choose the lowest energy; (2) if only one energy is in the visible range, choose that one; (3) if multiple peaks are in the visible range, choose the one with the highest oscillator strength. While the vertical excitation energy is not exactly analogous to *λ*_max,abs_ because it does not account for nuclear vibronic effects, it is a relatively cheap computational surrogate that should improve the capability of a model to predict *λ*_max,abs_.

### 2.3 Dye and solvent representations

We compared three representation methods for the dye molecules and four for solvents. Two of the dye representations were derived from the open-source Chemprop D-MPNN framework,^32^ and we compared these representations to the ChemFluor Functionalized Structure Descriptor (FSD) representation developed by Ju *et al.*^16^ The FSD representation is a composite fingerprint created by concatenating the E-state, CDK extended, and substructure presence and count fingerprints calculated by the PaDEL software^44^ through PaDELPy.^45^ Ju *et al.* found FSD to be superior in an extensive benchmark against other fixed fingerprint representations for predicting molecular absorption and emission peak energies. One Chemprop representation uses the D-MPNN framework “as is” to create a fingerprint embedding that is optimized for predicting absorption peak energies. The second Chemprop representation (which we call ChempropMultiFidelity) is similar to the first, but it uses a second Chemprop model trained on TD-DFT results to predict the TD-DFT peak vertical excitation energy and concatenates this predicted value onto the first Chemprop fingerprint embedding.

The four solvent representations compared herein are Morgan fingerprints, ChemFluor Comprehensive General Solvent Descriptors (CGSD), Minnesota solvent descriptors,^46^ and Chemprop D-MPNN embeddings (SolventMPNN). We calculated the Morgan fingerprints with a radius of 4 and 256 bits. The five CGSD descriptors (developed by Ju *et al.*^16^ in conjunction with the FSD dye representation) were extracted from the work of Reichardt^47^ and Catalán^48^ and represent the polarity (*E*_T_(30)), acidity (SA), basicity (SB), dipolarity (SdP), and polarizability (SP) of a solvent. We also matched solvents with their seven corresponding descriptors from the Minnesota Solvent Descriptor Database:^46^ index of refraction (*n*), Abraham's H-bond acidity (*α*), Abraham's H-bond basicity (*β*), surface tension (*γ*), dielectric constant (*ε*), aromaticity (*ϕ*), and electronegative halogenicity (*ψ*). The solvent D-MPNN embeddings were optimized using a separate D-MPNN alongside that of the dye; this approach was previously shown to be successful in predicting solvation free energies.^49^ This is also similar to the “direct-solvent” approach of Chen *et al.*,^50^ except that our dye and solvent D-MPNNs are not connected to one another until their embeddings are concatenated before the fully-connected feed-forward neural network (FFNN).

All dye–solvent pairs for which any of the above features could not be calculated were dropped from the dataset.

### 2.4 Models

**Fig. 1:**
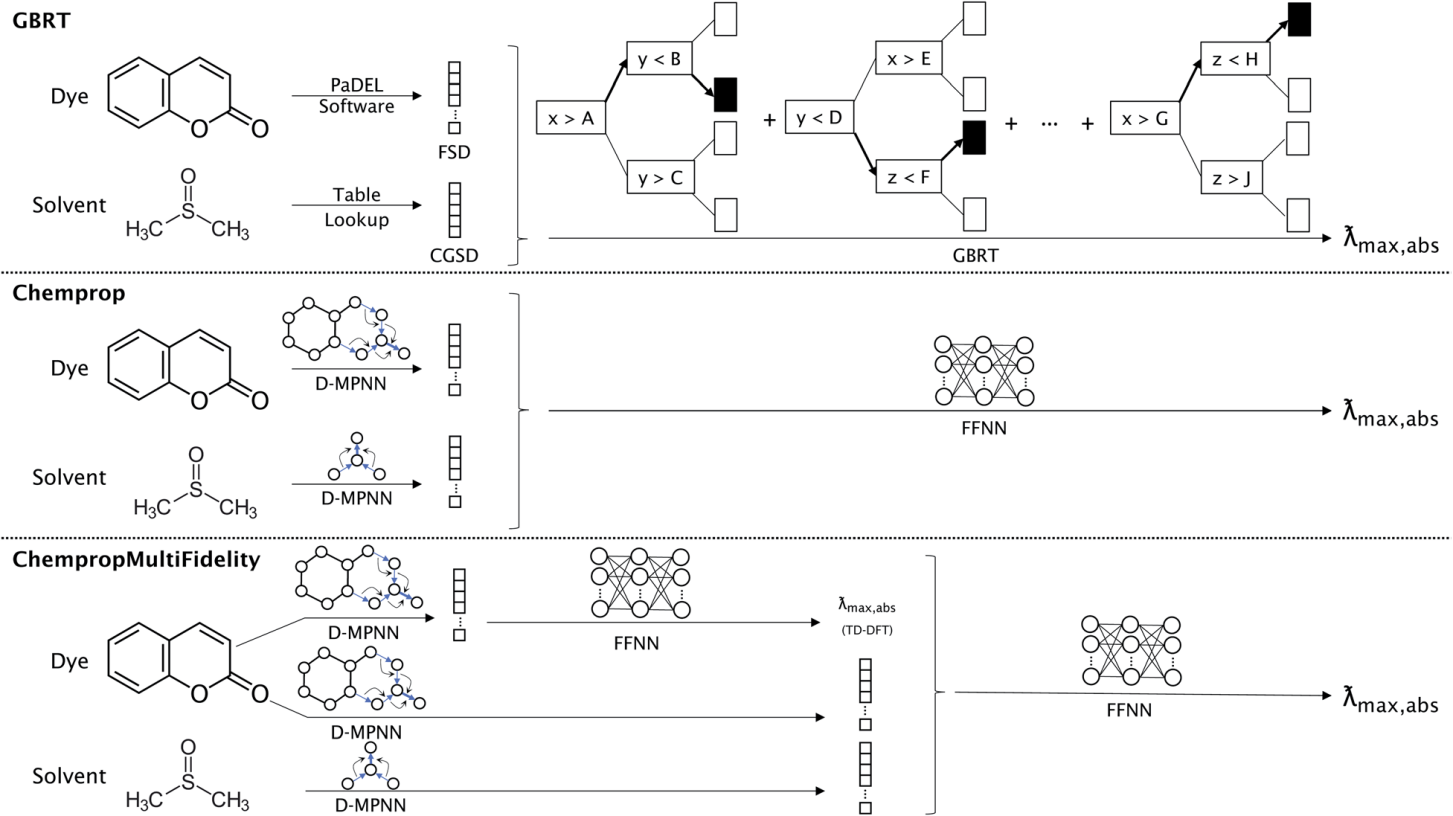
Model architectures for predicting experimental absorption peak in solvent. GBRT uses the PaDEL software and a table lookup to arrive at the FSD and CGSD, which are concatenated and used as input for a gradient boosted regression tree algorithm. Chemprop uses separate D-MPNN networks to obtain fingerprint embeddings for the dye and solvent, then concatenates these for input to a FFNN. The ChempropMultiFidelity model is similar to Chemprop, except with the addition of a secondary D-MPNN and FFNN that are trained to predict the absorption peak from TD-DFT data. This value is concatenated with the two fingerprint embeddings before being passed to a FFNN. There is no weight sharing between any of the D-MPNN networks. The Chemprop and ChempropMultiFidelity models can alternatively use Morgan fingerprints, Minnesota descriptors, or CGSD to represent the solvent in place of a D-MPNN; GBRT can use Morgan fingerprints or Minnesota descriptors as alternatives to CGSD.

We compare the performance of three types of models, each corresponding to one of the dye representations described above. The FSD representation was used with the gradient boosted regression tree (GBRT) algorithm^51^ as implemented by Ju *et al.*^16^ For both D-MPNN representations described above, the resulting fingerprint embedding is passed to a feed-forward neural network (FFNN) to accomplish the regression task. The three types of models are illustrated in Fig. 1. The ChempropMultiFidelity model is a variation of the hybrid physics-ML models reviewed by Jia *et al.*^52^

D-MPNN hyperparameters (including hidden sizes, numbers of layers, dropout, batch sizes, learning rates, and warm-up epochs) were tuned using SigOpt.^53^ The GBRT used the hyperparameters reported by Ju *et al.*, while all D-MPNN models used hyperparameters that were tuned on the same ChemFluor dataset used by Ju *et al.* The details of the model architectures, training, and predictions are given in the ESI.†

### 2.5 Train-validation-test splits

The type of splitting strategy used during the development of machine learning models is a crucial consideration when evaluating the accuracy and generalizability of a model.^27,28^ We compare three splitting strategies to illustrate this principle and to encourage the use of rigorous splitting strategies in subsequent work. In our regression task, the dye molecule and solvent molecule are both inputs to predict the peak wavelength of maximum absorption. The most naive splitting strategy, therefore, is to split randomly by dye–solvent pairs. If there are no duplicate measurements in a dataset, this splitting strategy makes it trivial to ensure that no pair is present in more than one of the training, validation, and test sets. Although the solvent effect can sometimes cause a substantial shift in the peak wavelength, the peaks measurements of the same dye in different solvents will be correlated. In other words, knowing the peak absorption wavelength of a particular dye in one solvent will likely improve the predictions of that same dye in a different solvent. This suggests a more rigorous splitting strategy where measurements are split by dye molecules rather than by dye–solvent pairs. This method ensures that any given dye molecule is restricted to either the training, validation, or test set, regardless of how many different solvent measurements are available. The final and most rigorous strategy discussed in this work is a scaffold split using the Bemis–Murcko scaffold^54^ implemented in RDKit through Chemprop. Scaffold splits ensure that any dye molecules that possess the same scaffold are restricted to a single set, which makes the regression task more challenging and provides a better evaluation of model generalizability. This splitting strategy is most reflective of performance on *de novo* design tasks with unseen chemistries. We used 80-10-10 training-validation-test proportions for all splits.

## 3 Results and discussion

We performed our analysis on a combination of five data sources, which comprised a total of 28 734 measurements (of which 26 623 were unique dye–solvent pairs). Of these 28 734, there were 1870 included in more than one data source. The combined dataset contained 15 157 unique dyes and 364 unique solvents. Ten of the solvents were used in 1000 or more measurements. The breakdown of our data by source and by solvent is represented in Tables 3 and S1.†

**Table 3:** Dataset composition by data source. The numbers for dye–solvent pairs correspond to the number of measurements after filtering. These numbers do not account for the aggregation of duplicate measurements either within or across datasets. There were 1870 measurements present in more than one data source

Dataset	Measurements
Deep4Chem	16 585
ChemFluor	4170
DyeAgg	3626
CDEx	1915
DSSCDB	2438
Total	28 734

**Fig. 2:**
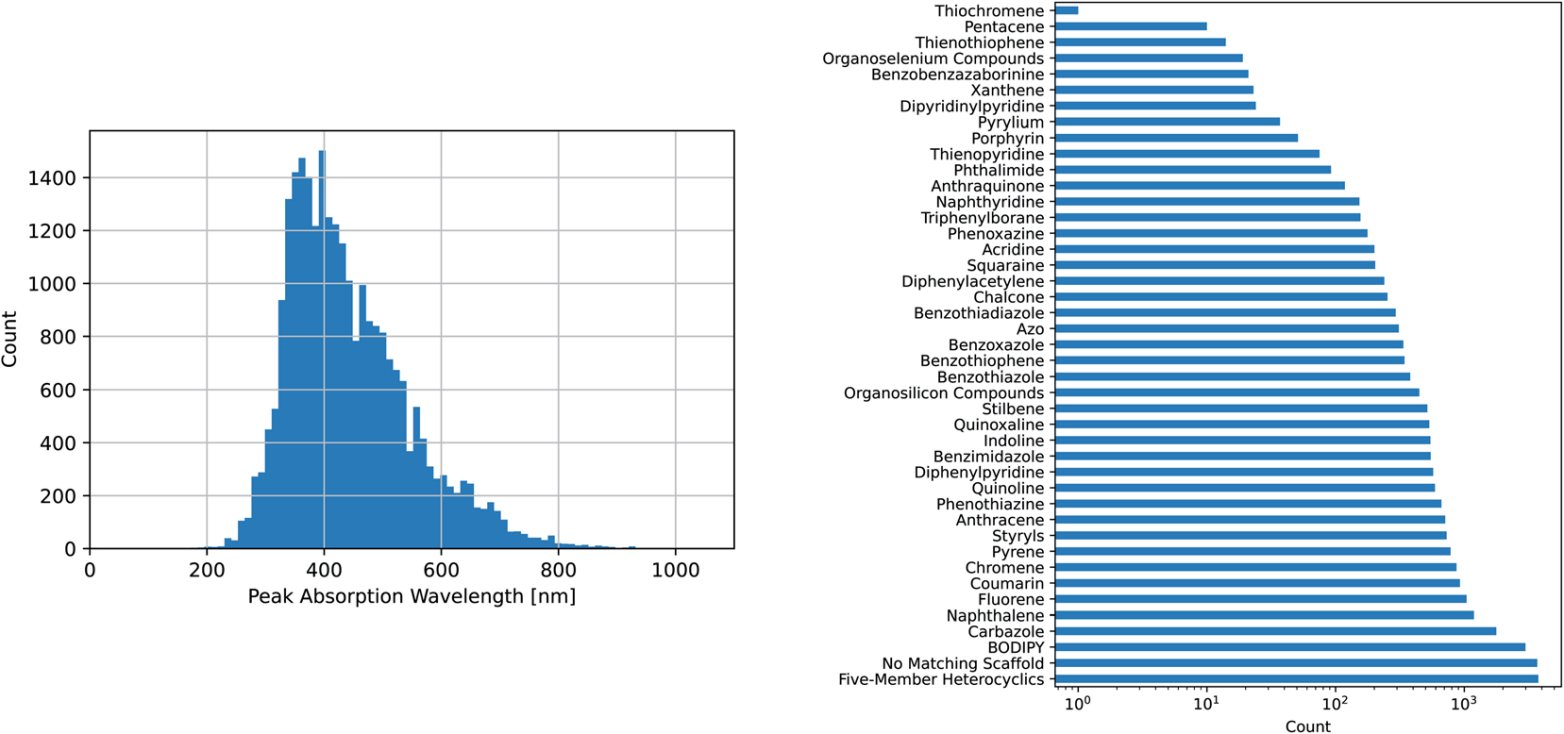
Dataset composition: peak location and common scaffold matches. (Left) The experimental peak wavelengths of maximum absorption from the combined dataset span the entire visible spectrum and extend into the infrared and ultraviolet, (Right) the combined dataset covers a wide variety of common dye scaffolds/families, as determined by SMARTS pattern matching.

The maximum absorption wavelengths of the dyes represented in our dataset cover the entire visible spectrum and extend into the ultraviolet and near-infrared regions. The molecules in the dataset include many of the common dye substructures and families. Fig. 2 illustrates the distribution of wavelengths and the prevalence of each substructure. The peak wavelength distributions of the individual datasets are different from that of the combined dataset and from each other, as shown in Fig. S1.†

**Fig. 3:**
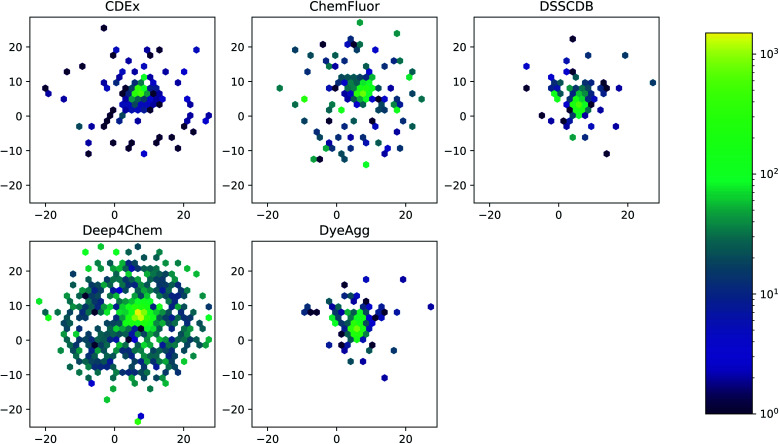
UMAP of Morgan fingerprints by data source. A UMAP dimensionality reduction on the Morgan fingerprints shows a difference in the coverage and density of each dataset in chemical space.

The five datasets differ in their coverage of chemical dye space, as shown in Fig. 3. The largest single data source (Deep4Chem) also has the most dense coverage of the chemical space. The smaller data sources, while covering a relatively large area of space, display more outliers that have few or zero close neighbors. Quantitatively, the mean Tanimoto similarities to nearest neighbor (based on RDKit fingerprints) are 0.760, 0.917, 0.937, 0.891, and 0.884 for the CDEx, ChemFluor, Deep4Chem, DSSCDB, and DyeAgg sets, respectively. Histograms of the pairwise Tanimoto similarities for each dataset are shown in Fig. S3.†

**Fig. 4:**
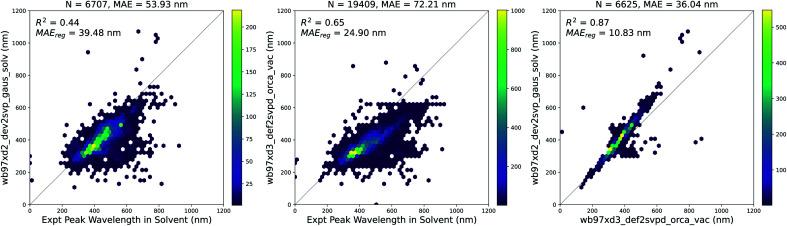
TD-DFT calculations in vacuum and solvent *vs.* experiments. (Left) Vertical excitation energy with maximum oscillator strength from solvent-corrected TD-DFT *versus* peak wavelength of maximum absorption from experiment, (Center) vertical excitation energy with maximum oscillator strength from vacuum TD-DFT *versus* peak wavelength of maximum absorption from experiment, (Right) vertical excitation energy with maximum oscillator strength from solvent-corrected TD-DFT *versus* vertical excitation energy with maximum oscillator strength from vacuum TD-DFT. In each plot, MAE_reg_ refers to the adjusted MAE value after performing a simple linear regression. Similar plots are shown in Fig. S4† with data points across all three plots corresponding to the same measurements. The linear regression equations for each plot above are as follows: (Left) *λ*_expt,solv_ = 1.69*λ*_tddft,solv_ − 238.24, (Center) *λ*_expt,solv_ = 1.82*λ*_tddft,vac_ − 226.82, and (Right) *λ*_tddft,solv_ = 1.18*λ*_tddft,vac_ − 32.86.

We used TD-DFT to calculate the vertical excitation energies for 10 947 molecules in vacuum and 6707 molecules with solvent corrections corresponding to the solvent measurements available in the experimental dataset. Since some molecules were measured in multiple solvents experimentally, the total number of experimental measurements with a corresponding vacuum TD-DFT calculation was 19 409. The results of these calculations are compared to experiments in Fig. 4. Solvent corrections applied to TD-DFT yield results that have a smaller error than vacuum calculations with respect to the experimental ground truth. However, after fitting a linear regression to both sets of calculations, the error for the vacuum calculations is lower. Therefore, this systematic error of the vacuum calculations makes it suitable to use the results of the vacuum calculations as features for building our models.

Although the combination of vacuum TD-DFT and a simple linear model performs well with respect to the experimental ground truth (MAE = 24.9 nm), the computational cost of TD-DFT may limit its applicability on large datasets. Nevertheless, this sets a baseline for the accuracy of computational methods in general when predicting this property.

We trained a D-MPNN and FFNN on 80-10-10 random splits of 28 772 vertical excitation energies from our full set of vacuum TD-DFT calculations. This model achieved a test MAE of 0.12 eV (14.99 nm), and the predictions are shown in Fig. S12.† This model became the auxiliary model used in the ChempropMultiFidelity approach for the remainder of this work.

**Fig. 5:**
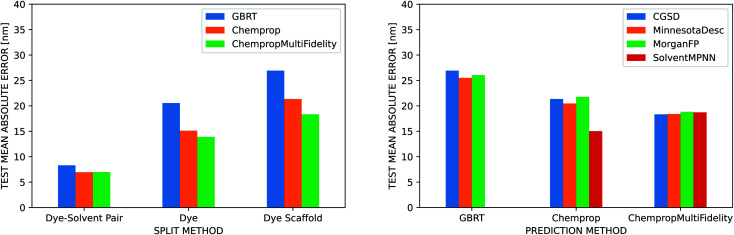
Dye and solvent representations. (Left) Comparison of three different molecular representations (GBRT, Chemprop, and ChempropMultiFidelity) across different methods for splitting into training, validation, and test sets on the Deep4Chem dataset. The CGSD is used to represent the solvent for all molecule representations. The ChempropMultiFidelity and Chemprop methods perform better than GBRT on all split types, and the difference in performance is more pronounced for the more rigorous split types. ChempropMultiFidelity performs best on the two more rigorous splits. (Right) Comparison of four different solvent representations (CGSD, Minnesota Solvent Descriptors, Morgan Fingerprint, and SolventMPNN) using scaffold splits of the Deep4Chem dataset. Regardless of which molecular representation is used, the CGSD and SolventMPNN representations outperform the others, albeit only slightly.

The three ML methods we benchmarked are all able to match or exceed the accuracy of the linear regression on the vacuum TD-DFT result, but their ability to do so is heavily influenced by the strategy used to split the data into training, validation, and test sets (Fig. 5). All three methods achieve a MAE under 10 nm when splitting by dye–solvent pair. For GBRT, this is similar to what Ju *et al.* observed in their predictions on the ChemFluor dataset (test set MAE of 10.46 nm). Performance worsens to MAEs of 13–21 nm when splitting by dye molecule and to 18–27 nm when splitting by dye scaffold. ChempropMultiFidelity achieved a test RMSE of 27.47 nm using the SolventMPNN representation and scaffold splits. This outperforms the graph-convolutional networks (GCN) approach of Joung *et al.*, who reported a test RMSE of 31.6 nm on random splits. Across all three splitting strategies, ChempropMultiFidelity and Chemprop perform better than GBRT. ChempropMultiFidelity performs better than Chemprop on the two more rigorous splitting strategies. This indicates that the Chemprop and ChempropMultiFidelity methods have generalizability superior to that of GBRT.

Ju *et al.* report that when they partitioned their data into training and test sets based on dye molecules rather than dye–solvent pairs, their test MAE in emission peak wavelength increased only slightly from 14.09 nm to 15.25 nm, but they did not report similar numbers for absorption predictions. Joung *et al.* do not report results for splitting by dye molecules, and neither report results when splitting by dye scaffold. Splitting more rigorously results in a wider error and ensemble variance distributions (Fig. S9†), but this is a better assessment of the ability of the model to generalize and is thus more reflective of performance for *de novo* design tasks. When using the random splitting strategy, dye molecules that appear in both the train and test set in different solvents have narrower error and ensemble variance distributions than those that only appear in the test set (Fig. S10†). As shown in Fig. S11,† we can also compare the splitting strategies by calculating the similarity (based on Morgan fingerprints or latent space coordinate) of each molecule in the test set to its nearest neighbor in the training set and plotting the test set error as a function of this similarity. When the similarity scores (which range from 0 to 1) are grouped into bins of size 0.1, this illustrates the error distributions as a function of similarity. The maximum prediction error should be lowest for the bin of test molecules that are most similar to the training set, but this was not true for the random splitting strategy. This indicates that the model may be relying too much on the training data and failing to learn the solvent effect. In contrast, the more rigorous splitting strategies exhibited the expected behavior.

The improved generalizability of the two D-MPNN approaches over the GBRT method may be the result of the automatically-learned dye representations. ChempropMultiFidelity outperforms Chemprop because in addition to this automatically-learned representation, it also incorporates additional physical knowledge through the inclusion of a predicted TD-DFT value in the learned embedding. It should be emphasized that this predicted TD-DFT value comes from an additional Chemprop model rather than an actual TD-DFT calculation, so there is no need to perform an additional calculation to predict on an unseen dye molecule. The ChempropMultiFidelity method achives a MAE of 18.3 nm on scaffold splits, an improvement over the TD-DFT plus linear regression approach at a fraction of the cost.

We compared our ChempropMultiFidelity approach of incorporating a TD-DFT feature predicted by an auxiliary model to using true TD-DFT values. As shown in Fig. S13,† the detrimental effect of the noise introduced using the D-MPNN TD-DFT feature may be outweighed by the dramatic savings in computational cost and time from no longer needing to perform a new TD-DFT calculation for an unseen molecule.

In our comparison of four different solvent representations, we found that none substantially and consistently outperform the others. It is necessary to represent the solvent in some way to achieve good predictions, but the CGSD, Minnesota descriptors, Morgan fingerprints, and SolventMPNN approaches all achieve similar results. The Morgan fingerprint and SolventMPNN approaches do, however, have the advantage that they are computable for any solvent since they are not restricted to look-up tables as are the CGSD and Minnesota methods.

**Fig. 6:**
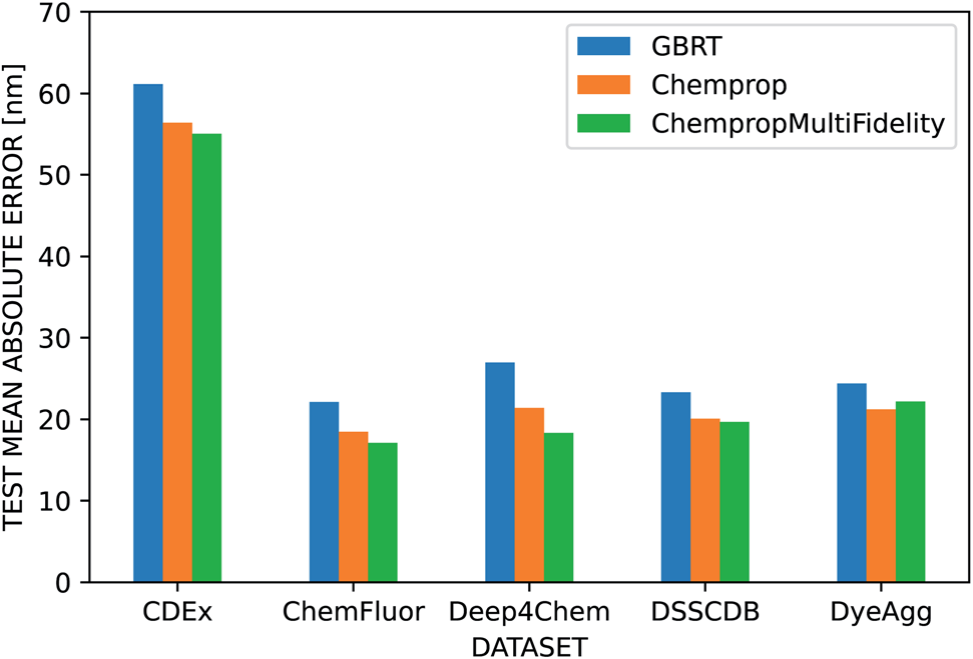
Performance of dye representations across datasets. Performance of three different molecular representations (GBRT, Chemprop, ChempropMultiFidelity) on several large, public datasets using scaffold splits and the CGSD solvent representation. Chemprop outperforms GBRT across all datasets. ChempropMultiFidelity is the best performer on all datasets except CDEx, for which all methods show substantially worse performance compared to the other datasets.

We used several additional datasets to further evaluate the performance of the three dye representations. ChempropMultiFidelity and Chemprop outperform GBRT on the ChemFluor, DSSCDB, and DyeAgg datasets and achieve MAEs of 17–23 nm, as shown in Fig. 6. All models perform substantially worse (55–62 nm) on the CDEx dataset, and the inclusion of the TD-DFT feature degrades performance. This may be a result of errors introduced by the automatic extraction method used to construct this dataset. The results of the aforementioned and additional experiments across different combinations of molecular and solvent representations and datasets are reported in terms of MAE, RMSE, and *R*^2^ in the ESI Fig. S5–S8 and Tables S4–S12.†

We explored the effect of combining datasets together and performed 5-fold cross-validation to draw more rigorous conclusions. After observing the exceptionally poor performance of all models on the CDEx dataset, we excluded it from the combined dataset. The GBRT method was excluded from this analysis to compare the Chemprop and ChempropMultiFidelity models using the D-MPNN solvent representation (which cannot be integrated into the GBRT method). ChempropMultiFidelity achieved a mean MAE, RMSE, and *R*^2^ of 27.78 nm, 47.13 nm, and 0.80, respectively. These scores outperformed Chemprop in all metrics, but there was overlap in the standard errors of all three scores. The complete results are shown in Table 4. The prediction errors on this larger, combined dataset are larger than those on the smaller datasets because of the different coverage of chemical space represented within each dataset. This is illustrated in Fig. S14–S18,† which show the results of training and predicting on different datasets. These results indicate that the ChemFluor and Deep4Chem datasets are relatively similar to one another, as are the DSSCDB and DyeAgg. Combining all four datasets together results in an inhomogeneous chemical space and thus lowers performance.

**Table 4:** Comparison of dye representations on a combined dataset. 5-Fold cross-validation on a dataset comprised of the union of the ChemFluor, Deep4Chem, DSSCDB, and DyeAgg dataset using scaffold splits and the D-MPNN solvent representation. The values represent the mean of the five cross-validation folds, while the error bars indicate the standard error

Model	MAE (nm)	RMSE (nm)	*R* ^2^
Chemprop	30.23 ± 5.62	52.08 ± 12.19	0.75 ± 0.08
ChempropMultiFidelity	27.78 ± 5.07	47.13 ± 11.10	0.80 ± 0.07

**Fig. 7:**
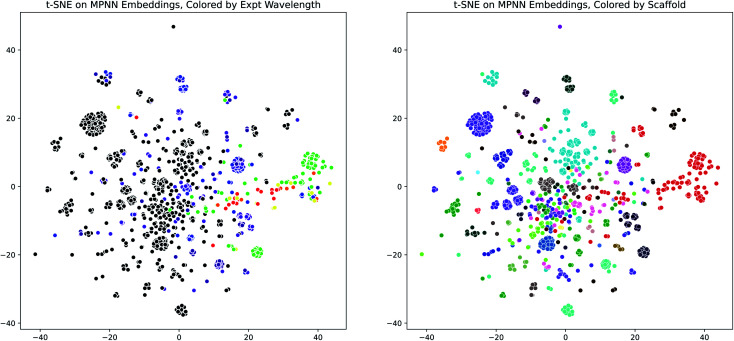
MPNN Embedding Interpretability through t-SNE. (Left) t-SNE plot (perplexity = 100) of molecule D-MPNN embeddings from the scaffold-split test set of the Deep4Chem dataset, using the Chemprop molecule representation and SolventMPNN solvent representation, colored by the experimental peak wavelength of maximum absorption. Colors outside the visible spectrum are shown as black. (Right) Same as (Left), but colored by dye family scaffold. The same plots are shown in Fig. S20† for a UMAP dimensionality reduction.

Having demonstrated the effectiveness of the D-MPNN models for modeling the peak wavelength of maximum absorption, we used dimensionality reduction to examine the interpretability of these models. We extracted the fingerprint embeddings from one of the Chemprop models and applied the t-SNE algorithm to reduce them to two dimensions. When these plots are colored by the experimental peak absorption wavelength and by the dye scaffolds present in the dataset, some patterns and clustering emerge, as shown in Fig. 7. The number of dye scaffolds that are known to absorb light at lower visible (green-violet) and ultraviolet wavelengths is much greater than those known to absorb in the red-yellow range of the spectrum. This is apparent in the t-SNE plots, as the red points clustered on the right of the scaffold plot (corresponding to the BODIPY dye family) comprise nearly all of the red-green points on the wavelength plot.

**Fig. 8:**
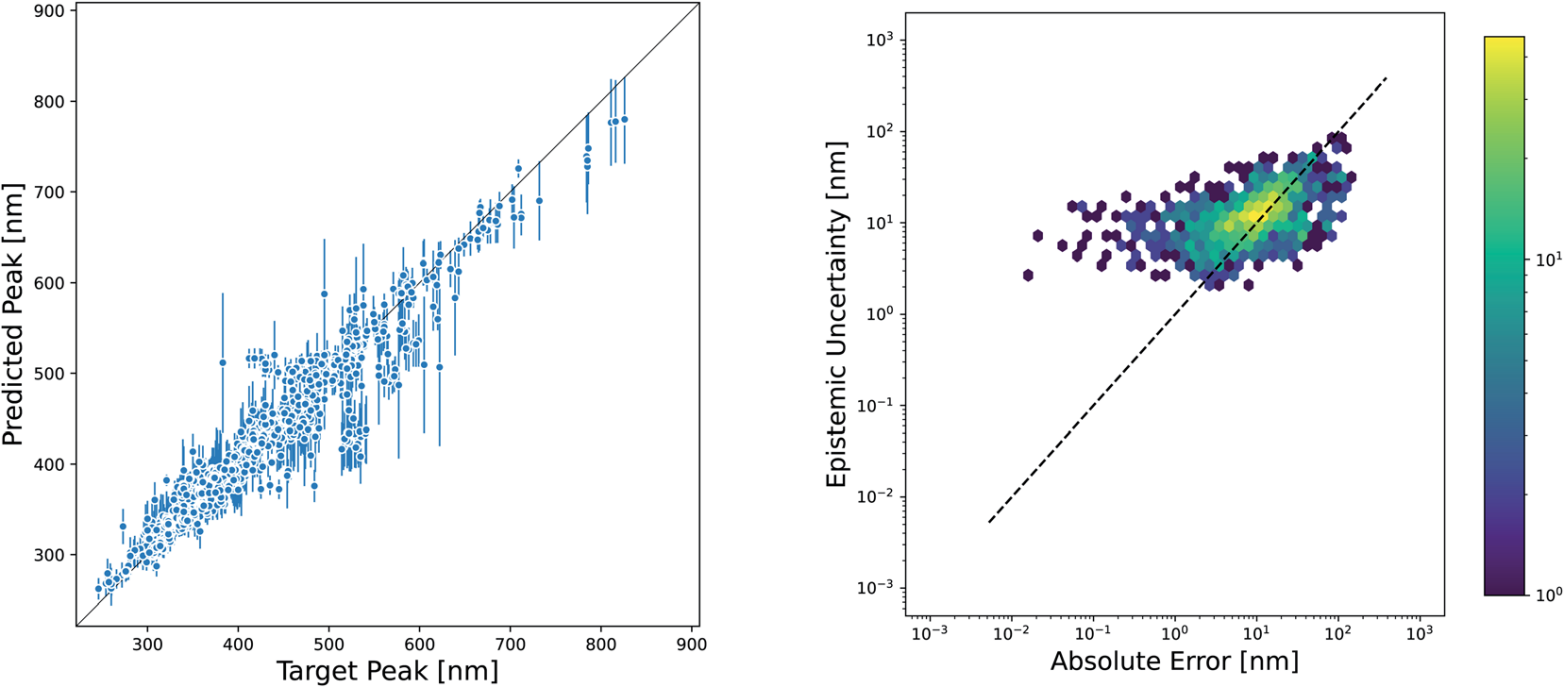
Uncertainty in D-MPNN models. (Left) The epistemic uncertainty in test set predictions of an D-MPNN model estimated from the ensemble variance using an ensemble of five models. Predictions are for scaffold splits of the Deep4Chem dataset using the Chemprop molecule representation and SolventMPNN solvent representation. Error bars represent the square root of the ensemble variance for consistency in units. Many poorly-predicted points have a high ensemble variance. (Right) Epistemic uncertainty compared to absolute error between prediction and experiment. The Spearman rank correlation is 0.52.

While the accuracy of a model is very important, the ability to quantify uncertainty in predictions can greatly increase model utility. The level of confidence in a prediction or set of predictions can be used to motivate the selection of candidates that are most likely to succeed in experimental validation or to inform the choice of new measurements that will improve the model through active learning techniques. There are a plethora of methods available for estimating the uncertainty in NN models, and reviews of these methods have not found one method that consistently performs others across datasets and evaluation metrics.^55–57^ However, one approach that is often used because of its ease of implementation is the ensemble variance as a measure of epistemic uncertainty. We evaluated the effectiveness of this method for quantifying the uncertainty in our Chemprop model. The parity plot in Fig. 8 shows that the variance of an ensemble of five models is indeed high for many of the predictions that fall far away from the parity line. However, closer examination by plotting the square root of the ensemble variance *versus* the absolute prediction error gives a much more sobering view of the effectiveness of this uncertainty metric. In fact, the Spearman rank correlation for this set of predictions and uncertainties is only 0.52, suggesting that one should not necessarily consider the rank order of the prediction uncertainties to be a good approximation of the rank ordering of the prediction errors.

## 4 Conclusions

We have leveraged several recently-published datasets to benchmark models in their prediction of the peak wavelength of maximum absorption for dye molecules. Our results showed that D-MPNN models outperformed the best known fixed-fingerprint regression tree method, and the performance gain was more pronounced when we used more rigorous splitting strategies to evaluate the generalizability of the models to unseen chemistries. We also developed a multi-fidelity method for incorporating data from TD-DFT calculations to improve the accuracy of experimental predictions. Vertical excitation energies from gas-phase TD-DFT calculations have a good linear correlation with the experimental peak positions (MAE = 24 nm) of dyes (measured in various solvents). TD-DFT PCM calculations in the solvents do not correlate as consistently with the experimental data.

Our best method (ChempropMultiFidelity) included a model trained on the results of previous TD-DFT calculations, and we used the predictions of this model as inputs to a second model that accounted for the solvent to predict the experimental peak wavelength. This multi-fidelity approach improved the model generalizability and improved performance for more rigorous splitting strategies. Our best model achieved a MAE of less than 7 nm on a held-out test set from a random split of dye–solvent pairs in the Deep4Chem dataset, and near 14 nm and 19 nm when splitting by dye molecule and dye scaffold, respectively. This is substantially better than the predictions of TD-DFT calculations alone, and at much lower cost. Our multi-fidelity approach has the advantage that the lower-fidelity data can cover a larger area of chemical space than the higher-fidelity data. Future work should compare this approach to additional methods for training ML models on multi-fidelity data, such as transfer learning (*e.g.* Fig. S19†), imputation, Δ-ML, and multi-target weighted-loss-function approaches.

D-MPNN approaches perform well across many of the largest publicly-available datasets of the peak absorption wavelength. Additionally, the ChempropMultiFidelity model outperformed Chemprop on a union of the four largest datasets. It achieved a MAE of 27.78 ± 5.07 nm with 5-fold cross validation on scaffold splits of this combined dataset.

We also compared several solvent representations and found that CGSD, Morgan fingerprints, Minnesota descriptors, and D-MPNN fingerprint representations all performed similarly. The Morgan fingerprint and D-MPNN approaches may be advantageous, however, because they can be applied to any solvent because they are not restricted to look-up tables. Future work could also use nearest-neighbor imputation techniques to estimate CGSD or Minnesota descriptors that are not present in the look-up tables.

We demonstrated the qualitative interpretability of our D-MPNN models using dimensionality reduction on their latent space fingerprints, which showed some clustering based on dye scaffold and observed peak wavelength. We also showed that although ensemble variance can be used as a measure of the epistemic uncertainty in our D-MPNN model predictions, and in this case the ensemble errors are comparable in magnitude to the prediction errors, these uncertainties are not necessarily well-correlated with true prediction error on these datasets.

This work is a step toward methods to predict full absorption and emission spectra, and it can enable more rapid design of dye molecules with targeted optical properties.

## Data availability

All code to reproduce our workflow and figures and all data including TD-DFT calculation results is available at https://doi.org/10.5281/zenodo.5773155. To make predictions using Chemprop and ChempropMultiFidelity models, you can use the UVVisML tool at https://github.com/learningmatter-mit/uvvisml.

## Author contributions

R. G.-B. conceived the project. K. P. G. performed the simulations, wrote the computer code, analyzed the data, and wrote the first manuscript draft. W. H. G. and R. G.-B. supervised the research and edited the manuscript.

## Conflicts of interest

There are no conflicts to declare.

## Supplementary Material

SC-013-D1SC05677H-s001
