# Supplementary material for: Multi-fidelity prediction of molecular optical peaks with deep learning
Source: Chem Sci. 2022 Jan 4;13(4):1152–62. doi: 10.1039/d1sc05677h (PMC8790778; doi:10.1039/d1sc05677h)
Supplement: SC-013-D1SC05677H-s001 [file SC-013-D1SC05677H-s001.pdf]

Electronic Supplementary Material (ESI) for Chemical Science.
This journal is © The Royal Society of Chemistry 2022

Supporting Information

Multi-fidelity prediction of molecular optical peaks with deep learning

Kevin P. Greenman,† William H. Green,† and Rafael Gómez-Bombarelli*,‡

†*Department of Chemical Engineering, MIT, Cambridge, MA*

‡*Department of Materials Science and Engineering, MIT, Cambridge, MA*

E-mail: rafagb@mit.edu

Code and Data Availability

All code to reproduce our workflow and figures and all data including TD-DFT calculation results is available at `https://doi.org/10.5281/zenodo.5500428`. To make predictions using Chemprop and ChempropMultiFidelity models, you can use the UVVisML tool at `https://github.com/learningmatter-mit/uvvisml`.

Note on CGSD Solvent Descriptors

We identified two transcription errors by Ju et al. in the CGSD solvent descriptors (available from `https://figshare.com/articles/dataset/ChemFluor/12110619/3`) used to train their GBRT models.[1]

- In row 22 of `Solvent_Descriptors.xlsx`, the $E_T(30)$ value for 1-methyl-2-pyrrolidinone should be 42.2 rather than 48 according to entry no. 284 of Table 2 of Reichardt's work.[2]

- In row 24 of Solvent_Descriptors.xlsx, the SP, SdP, SA, and SB values for N-methylformamide are taken from entry no. 29 (N,N-dimethylformamide) of Table 2 of Catalán's work.[3] No entry exists for N-methylformamide.

We corrected these errors in our file data_solvents/chemfluor_cgsd_solvent_db.csv. This file contains more solvents than the original ChemFluor file because we parsed the entirety of the entries in Reichardt's and Catalán's works to have coverage over as many solvents as possible (rather than restricting ourselves to those in the ChemFluor dataset).

Dataset Composition

Table S1: **Dataset Composition by Solvent.** Ten of the 364 unique solvents appeared in more than 1,000 measurements.

Solvent Name	Solvent SMILES	Number of Measurements
Other	-	5653
Dichloromethane	ClCCl	5621
Tetrahydrofuran	C1CCOC1	3328
Chloroform	ClC(Cl)Cl	2423
Acetonitrile	CC#N	2343
Ethanol	CCO	1824
Methylbenzene	Cc1ccccc1	1793
Methanol	CO	1758
DMSO	CS(C)=O	1637
Water	O	1181
Dimethylformamide	CN(C)C=O	1173

Datasets Comparison

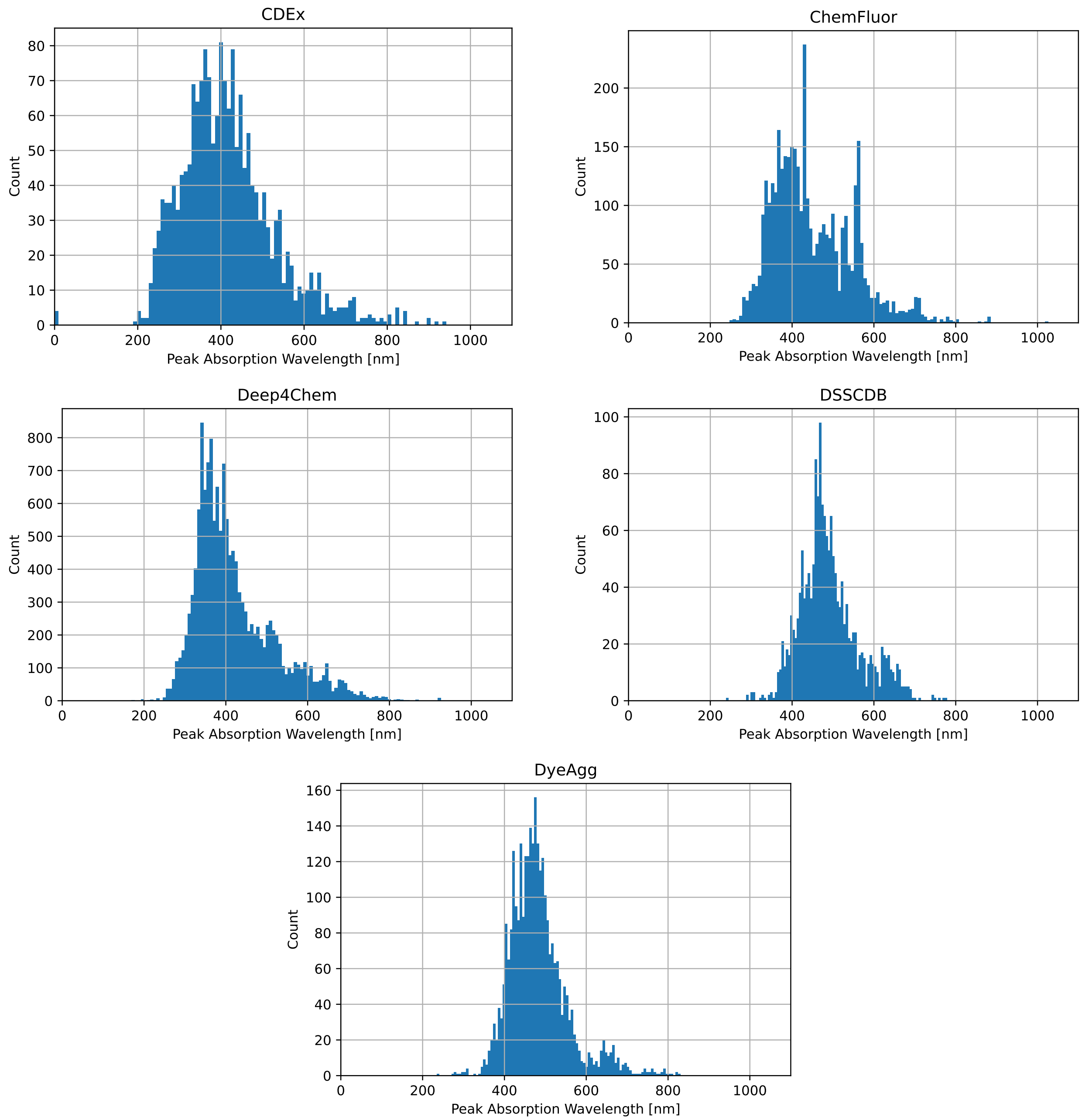

Figure S1: **Dataset Comparison.** The five datasets differ in their coverage of chemical space, which results in different distributions of peak absorption wavelength.

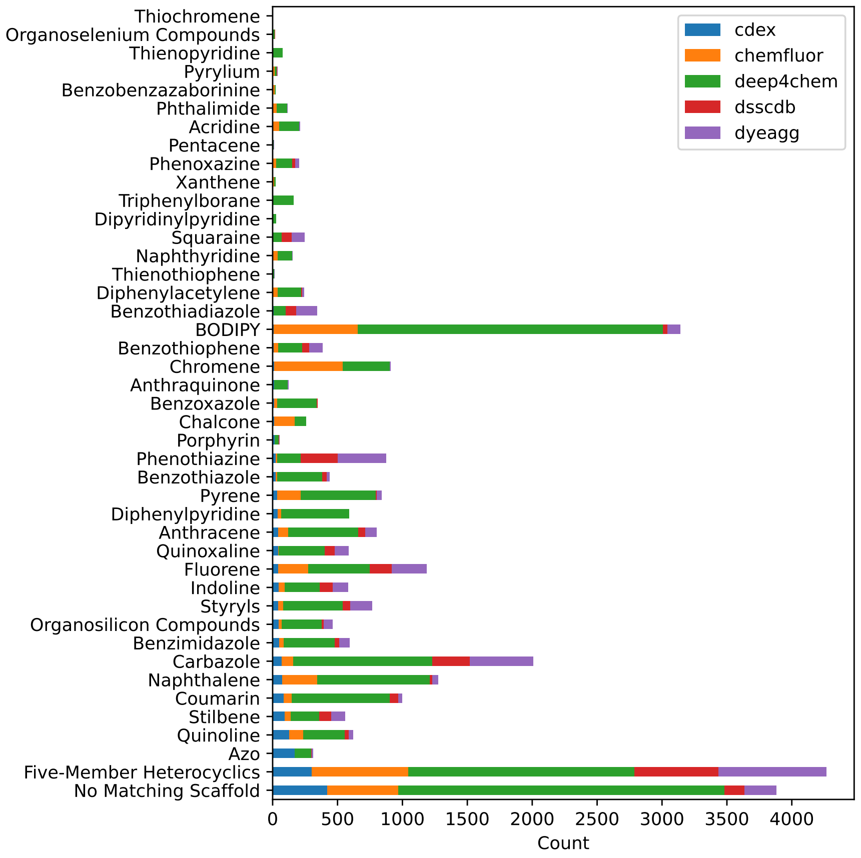

Figure S2: **Dataset Comparison by Scaffold.** The presence of each scaffold varies across datasets.

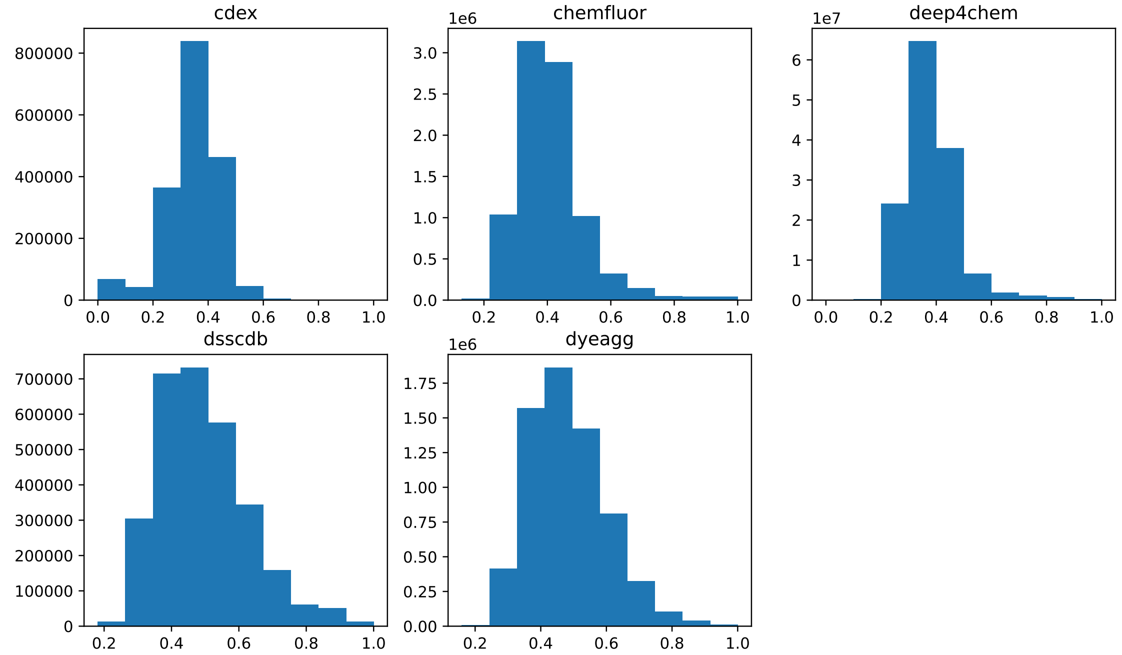

Figure S3: **Dataset self-similarity.** Count vs. pairwise Tanimoto similarity for each data source. Tanimoto similarity is computed on RDKit fingerprints.

Model Hyperparameters

All models for comparing different molecular and solvent representations and datasets, as well as the transfer learning baseline, were ensembles of 5 models with different random initializations. The TD-DFT imputation results in Figure S13 used a single model, and the cross-validation study reported in Table **??** used a single model for each fold.

Table S2: **Experimental Chemprop Model Hyperparameters**. Available as a JSON `sigopt/sigopt_chemprop_best_hyperparams.json` in the archive at `https://doi.org/10.5281/zenodo.5500428`.

Parameter	Value
batch size	10
depth	5
dropout	0.0723413445303434
FFN hidden size	444
FFN number of layers	3
final LR	0.0006545906904
hidden size	482
initial LR	0.000001003415726
max LR	0.0009989252530750487
warmup epochs	6

Table S3: **TD-DFT Chemprop Model Hyperparameters**. Available as a JSON `sigopt/sigopt_chemprop_tddft_best_hyperparams.json` in the archive at `https://doi.org/10.5281/zenodo.5500428`.

Parameter	Value
batch size	93
depth	6
dropout	0.05957668605969951
FFN hidden size	768
FFN number of layers	2
final LR	0.0001621326247
hidden size	1116
initial LR	0.0008541863938638898
max LR	0.0008541863938638898
warmup epochs	4

TD-DFT Results

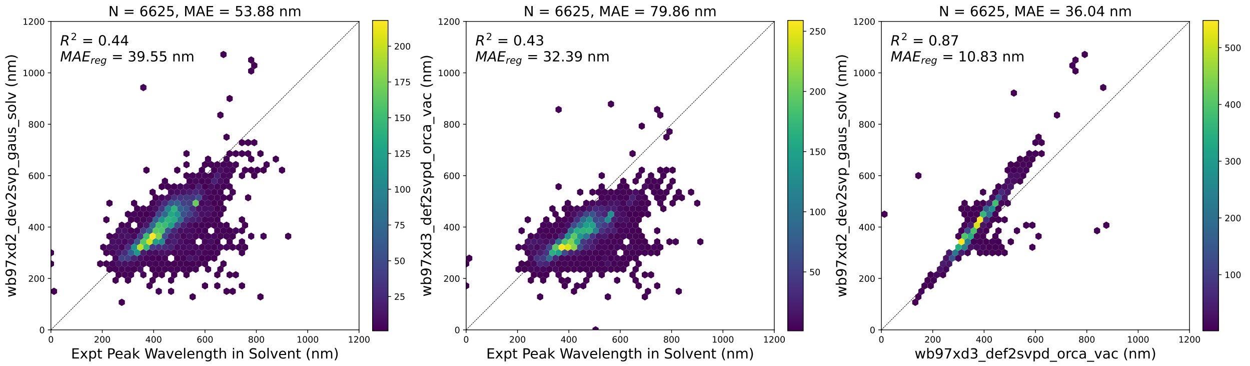

Figure S4: **TD-DFT Calculations in Vacuum and Solvent vs. Experiments.** (Left) Vertical excitation energy with maximum oscillator strength from solvent-corrected TD-DFT versus peak wavelength of maximum absorption from experiment, (Center) Vertical excitation energy with maximum oscillator strength from vacuum TD-DFT versus peak wavelength of maximum absorption from experiment, (Right) Vertical excitation energy with maximum oscillator strength from solvent-corrected TD-DFT versus Vertical excitation energy with maximum oscillator strength from vacuum TD-DFT. In each plot, MAE_{reg} refers to the adjusted MAE value after performing a simple linear regression. The data points across all three plots correspond to the same measurements. The linear regression equations for each plot above are as follows: (Left) $\lambda_{expt,solv} = 1.70\lambda_{tddft,solv} - 237.27$, (Center) $\lambda_{expt,solv} = 2.18\lambda_{tddft,vac} - 358.25$, and (Right) $\lambda_{tddft,solv} = 1.18\lambda_{tddft,vac} - 32.86$.

RMSE Bar Plots

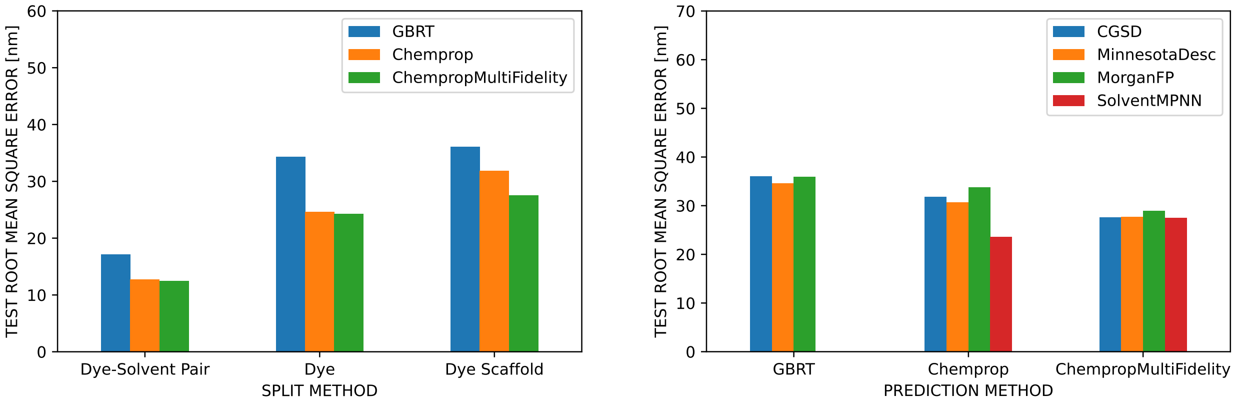

Figure S5

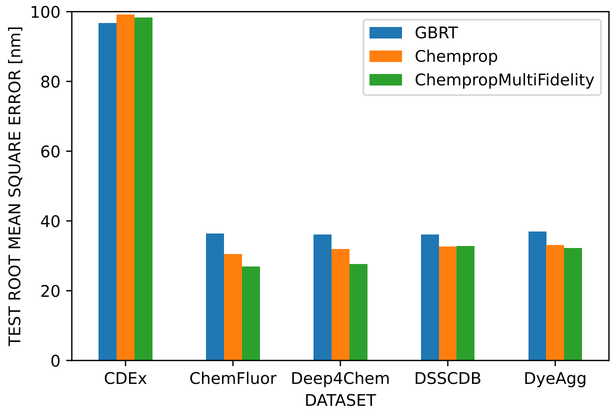

Figure S6

R2 Bar Plots

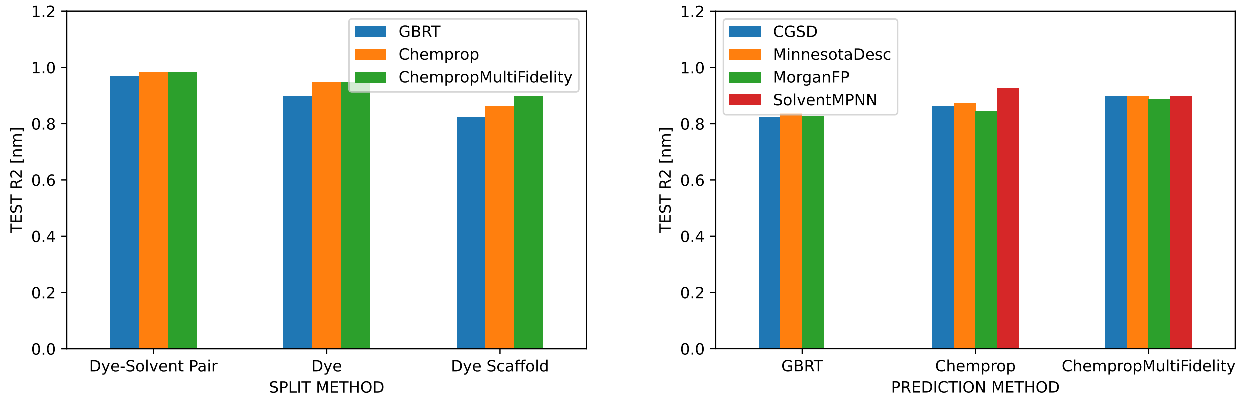

Figure S7

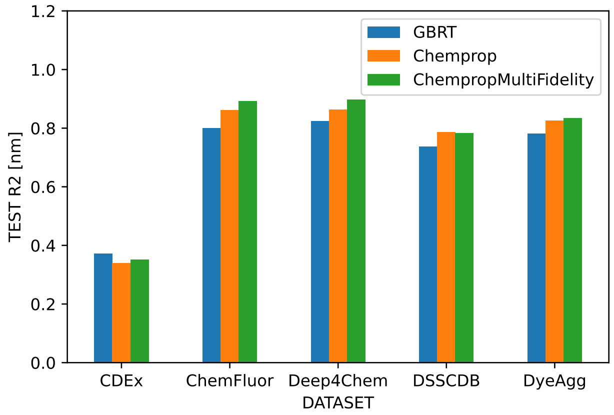

Figure S8

MAE, RMSE, and R^2 Tables

Table S4: Molecule Representations - MAE (Deep4Chem Dataset)

GBRT	Chemprop	ChempropMultiFidelity	Solvent Rep.	Split Type
8.30	6.95	6.97	CGSD	random
8.31	7.32	7.08	Minnesota	random
8.98	8.27	7.89	Morgan	random
NaN	7.00	6.99	MPNN	random
20.55	15.12	13.90	CGSD	group_by_smiles
20.90	14.96	14.17	Minnesota	group_by_smiles
20.79	15.01	13.77	Morgan	group_by_smiles
NaN	14.29	14.16	MPNN	group_by_smiles
26.94	21.35	18.33	CGSD	scaffold
25.51	20.47	18.40	Minnesota	scaffold
26.05	21.77	18.84	Morgan	scaffold
NaN	15.03	18.72	MPNN	scaffold

Table S5: Molecule Representations - RMSE (Deep4Chem Dataset)

GBRT	Chemprop	ChempropMultiFidelity	Solvent Rep.	Split Type
17.18	12.74	12.49	CGSD	random
16.65	12.90	12.54	Minnesota	random
17.56	14.24	13.68	Morgan	random
NaN	12.93	12.70	MPNN	random
34.29	24.60	24.24	CGSD	group_by_smiles
34.36	23.94	25.24	Minnesota	group_by_smiles
34.11	25.31	23.37	Morgan	group_by_smiles
NaN	24.01	25.06	MPNN	group_by_smiles
36.08	31.87	27.57	CGSD	scaffold
34.58	30.74	27.70	Minnesota	scaffold
35.92	33.77	28.91	Morgan	scaffold
NaN	23.60	27.47	MPNN	scaffold

Table S6: Molecule Representations - R^2 (Deep4Chem Dataset)

GBRT	Chemprop	ChempropMultiFidelity	Solvent Rep.	Split Type
0.97	0.98	0.98	CGSD	random
0.97	0.98	0.98	Minnesota	random
0.97	0.98	0.98	Morgan	random
NaN	0.98	0.98	MPNN	random
0.90	0.95	0.95	CGSD	group_by_smiles
0.90	0.95	0.94	Minnesota	group_by_smiles
0.90	0.94	0.95	Morgan	group_by_smiles
NaN	0.95	0.94	MPNN	group_by_smiles
0.82	0.86	0.90	CGSD	scaffold
0.84	0.87	0.90	Minnesota	scaffold
0.83	0.85	0.89	Morgan	scaffold
NaN	0.92	0.90	MPNN	scaffold

Table S7: Solvent Representations - MAE (Deep4Chem Dataset)

CGSD	MinnesotaDesc	MorganFP	SolventMPNN	Model	Split Type
8.30	8.31	8.98	NaN	GBRT	random
6.95	7.32	8.27	7.00	Chemprop	random
6.97	7.08	7.89	6.99	ChempropMultiFidelity	random
20.55	20.90	20.79	NaN	GBRT	group_by_smiles
15.12	14.96	15.01	14.29	Chemprop	group_by_smiles
13.90	14.17	13.77	14.16	ChempropMultiFidelity	group_by_smiles
26.94	25.51	26.05	NaN	GBRT	scaffold
21.35	20.47	21.77	15.03	Chemprop	scaffold
18.33	18.40	18.84	18.72	ChempropMultiFidelity	scaffold

Table S8: Solvent Representations - RMSE (Deep4Chem Dataset)

CGSD	MinnesotaDesc	MorganFP	SolventMPNN	Model	Split Type
17.18	16.65	17.56	NaN	GBRT	random
12.74	12.90	14.24	12.93	Chemprop	random
12.49	12.54	13.68	12.70	ChempropMultiFidelity	random
34.29	34.36	34.11	NaN	GBRT	group_by_smiles
24.60	23.94	25.31	24.01	Chemprop	group_by_smiles
24.24	25.24	23.37	25.06	ChempropMultiFidelity	group_by_smiles
36.08	34.58	35.92	NaN	GBRT	scaffold
31.87	30.74	33.77	23.60	Chemprop	scaffold
27.57	27.70	28.91	27.47	ChempropMultiFidelity	scaffold

Table S9: Solvent Representations - R^2 (Deep4Chem Dataset)

CGSD	MinnesotaDesc	MorganFP	SolventMPNN	Model	Split Type
0.97	0.97	0.97	NaN	GBRT	random
0.98	0.98	0.98	0.98	Chemprop	random
0.98	0.98	0.98	0.98	ChempropMultiFidelity	random
0.90	0.90	0.90	NaN	GBRT	group_by_smiles
0.95	0.95	0.94	0.95	Chemprop	group_by_smiles
0.95	0.94	0.95	0.94	ChempropMultiFidelity	group_by_smiles
0.82	0.84	0.83	NaN	GBRT	scaffold
0.86	0.87	0.85	0.92	Chemprop	scaffold
0.90	0.90	0.89	0.90	ChempropMultiFidelity	scaffold

Table S10: Datasets - MAE (CGSD Solvent Representation)

GBRT	Chemprop	ChempropMultiFidelity	Dataset	Split Type
61.10	56.40	55.04	CDEx	scaffold
22.08	18.48	17.10	ChemFluor	scaffold
26.94	21.35	18.33	Deep4Chem	scaffold
23.27	20.08	19.69	DSSCDB	scaffold
24.36	21.18	22.14	DyeAgg	scaffold

Table S11: Datasets - RMSE (CGSD Solvent Representation)

GBRT	Chemprop	ChempropMultiFidelity	Dataset	Split Type
96.70	99.16	98.27	CDEx	scaffold
36.36	30.39	26.82	ChemFluor	scaffold
36.08	31.87	27.57	Deep4Chem	scaffold
36.07	32.59	32.79	DSSCDB	scaffold
36.93	33.01	32.11	DyeAgg	scaffold

Table S12: Datasets - R^2 (CGSD Solvent Representation)

GBRT	Chemprop	ChempropMultiFidelity	Dataset	Split Type
0.37	0.34	0.35	CDEx	scaffold
0.80	0.86	0.89	ChemFluor	scaffold
0.82	0.86	0.90	Deep4Chem	scaffold
0.74	0.79	0.78	DSSCDB	scaffold
0.78	0.83	0.83	DyeAgg	scaffold

Error and Uncertainty Distribution Plots

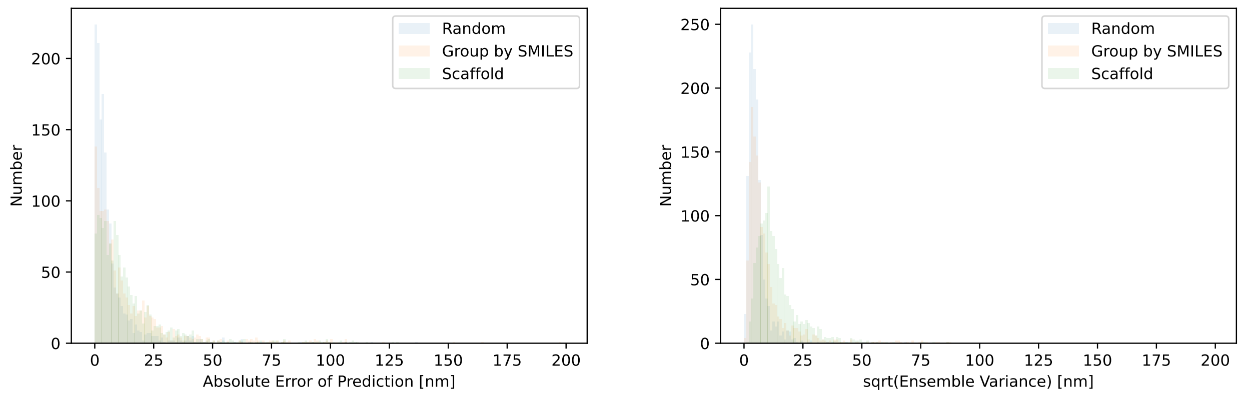

Figure S9: **Error and Uncertainty Distributions for 3 Split Types.** The error and uncertainty distributions both spread out as the splits become more rigorous from random to group by SMILES to scaffold.

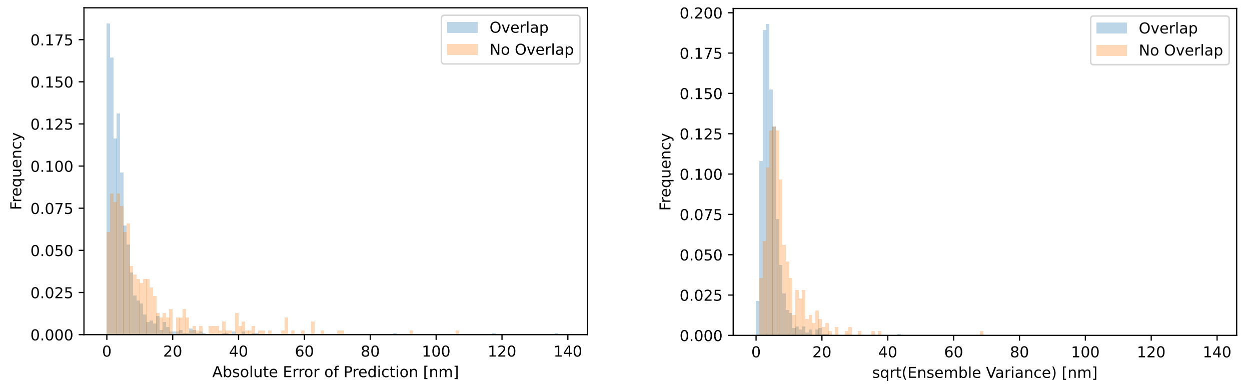

Figure S10: **Error and Uncertainty Distributions of Random Splits.** Using random splits, many dye molecules appear in both the train and test sets because they were measured in different solvents. For such molecules (”overlap”), the error and uncertainty distributions are tighter than for those molecules without overlap in train and test sets.

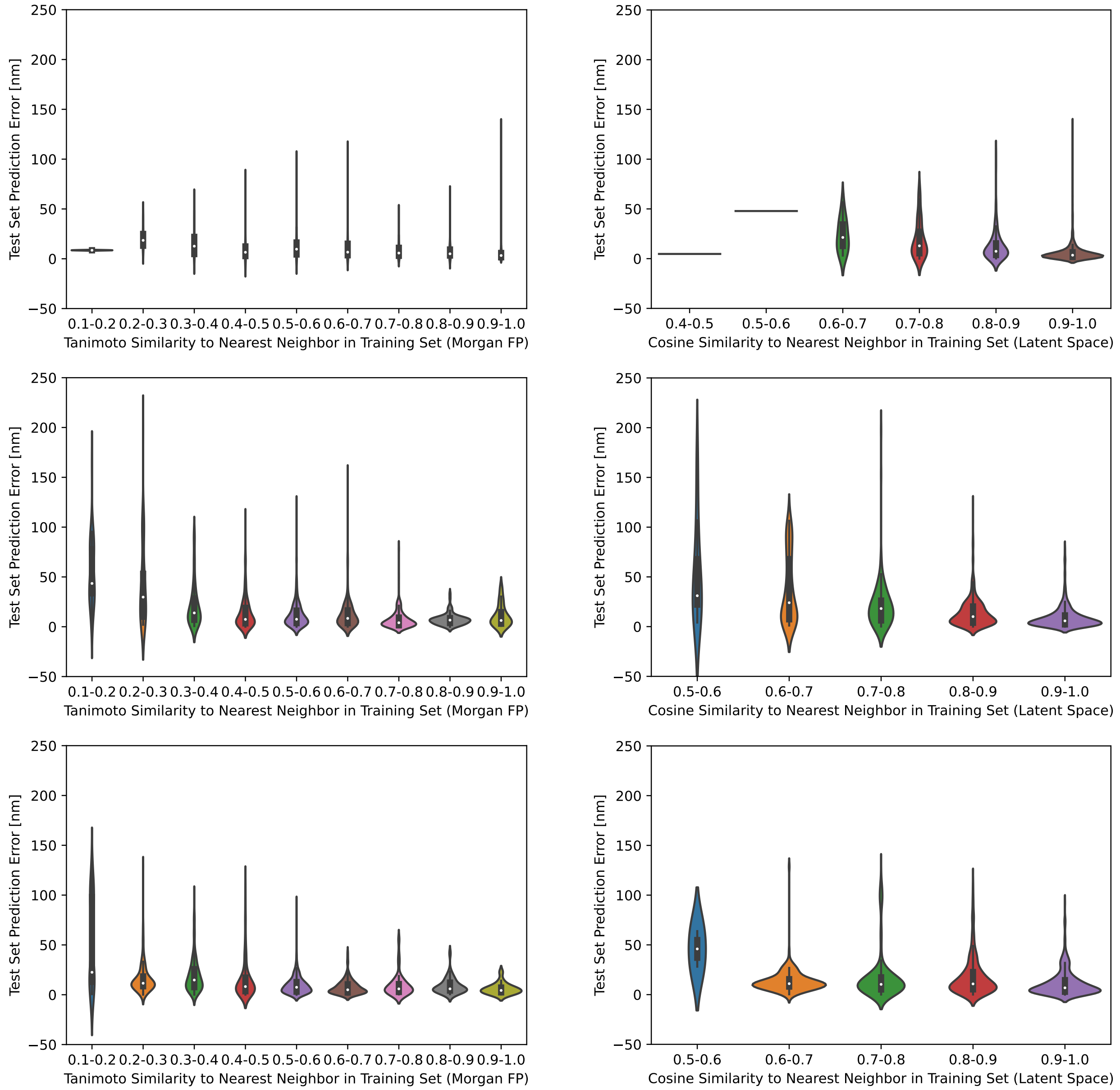

Figure S11: **Error distributions as a function of similarity to training set.** (Top) random splits; (Middle) group by SMILES splits; (Bottom) scaffold splits. As similarity to the nearest neighbor in the training set increases, we expect that the maximum of the prediction errors will decrease. This is mostly true for the more rigorous split types, but nearly the opposite is true for random splits.

TD-DFT Model Performance

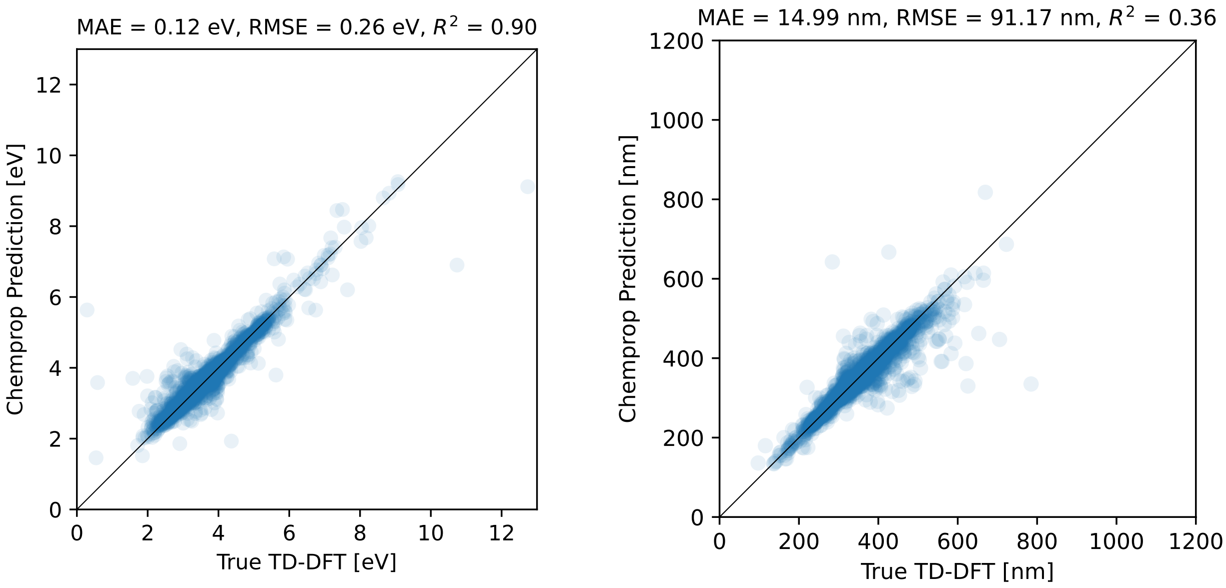

Figure S12: **Parity plot of Chemprop TD-DFT Predictions vs. True TD-DFT Results**. (Left) Units of eV; (Right) Units of nm. Both plots represent the exact same data (predictions of a model trained on data in eV). The TD-DFT model (a Chemprop D-MPNN and FFNN) used an ensemble size of 1 and was trained on 80-10-10 random splits of 28,772 vertical excitation energies from our full set of vacuum TD-DFT calculations. The large RMSE and low R^2 in nanometers is a result of the few outliers with very low true TD-DFT values (these values are visible on the left-most part of the eV plot with true TD-DFT values less than 1 eV, but they exceed the upper limit on the x-axis in the nm plot).

TD-DFT as a Feature

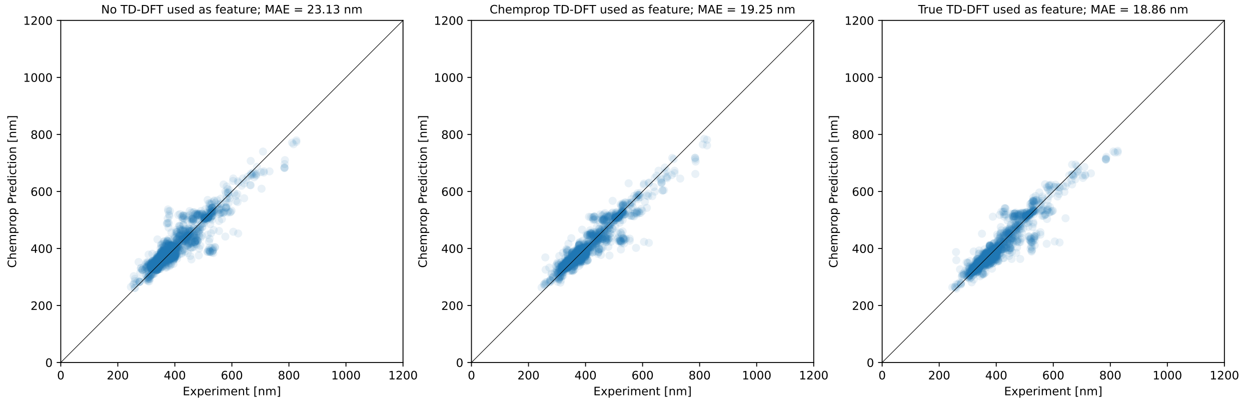

Figure S13: **Trade-Off Between True TD-DFT and Chemprop-Predicted TD-DFT as a Feature.** Prediction using (Left) no additional features, (Center) the TD-DFT value predicted by an auxiliary Chemprop D-MPNN model as a feature, or (Right) the true TD-DFT value as a feature. All models used a D-MPNN solvent representation, scaffold splits of the Deep4Chem dataset, and an ensemble size of 1. Using true TD-DFT values results in better performance (MAE = 18.86 nm) than using TD-DFT values predicted by the Chemprop D-MPNN (MAE = 19.25 nm), but the D-MPNN method retains most of the benefit over using no TD-DFT feature at all (23.13 nm).

Comparing Predictions Across Data Sources

All models in this section used the CGSD solvent representation. Each data source (CDEx, ChemFluor, Deep4Chem, DSSCDB, and DyeAgg) was split by scaffolds, but the training set from one data source may have scaffolds that overlap with those in the test set of another data source

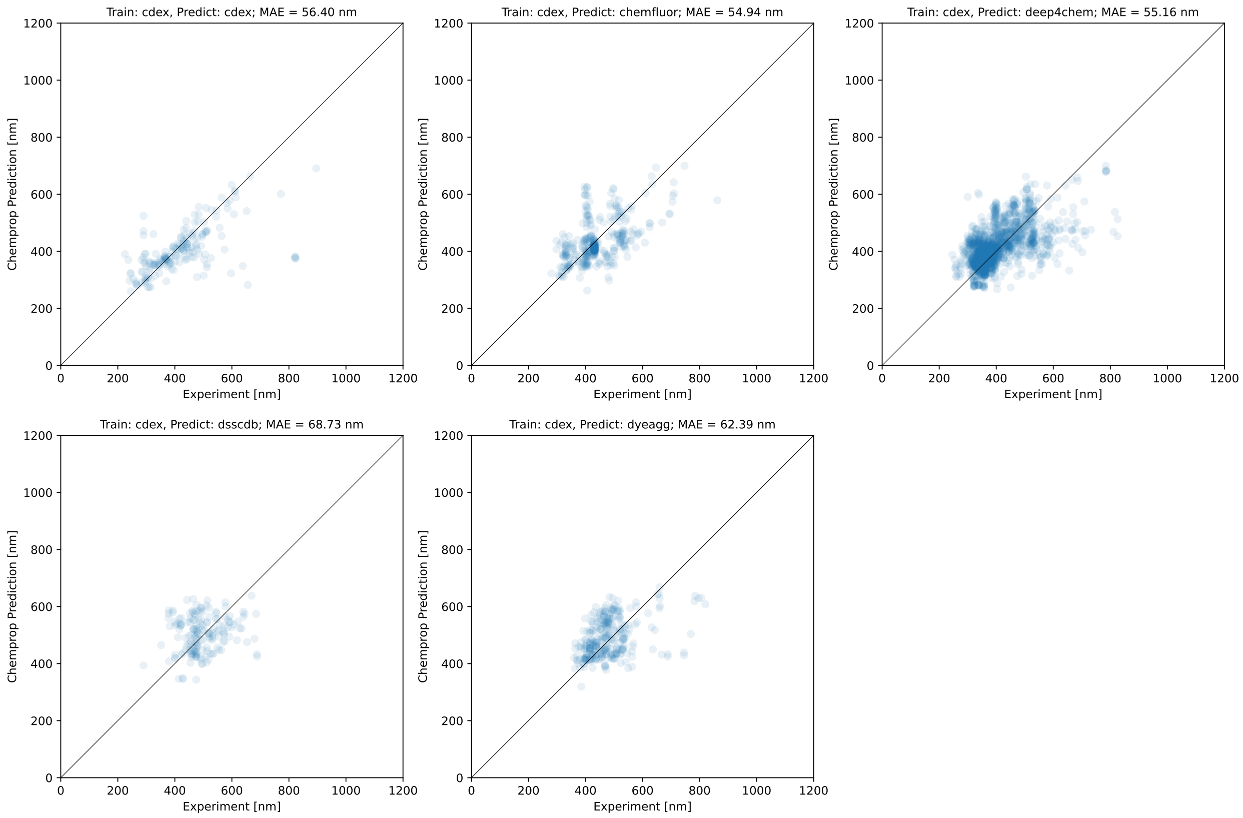

Figure S14: **Predictions on all datasets after training on CDEx dataset.**

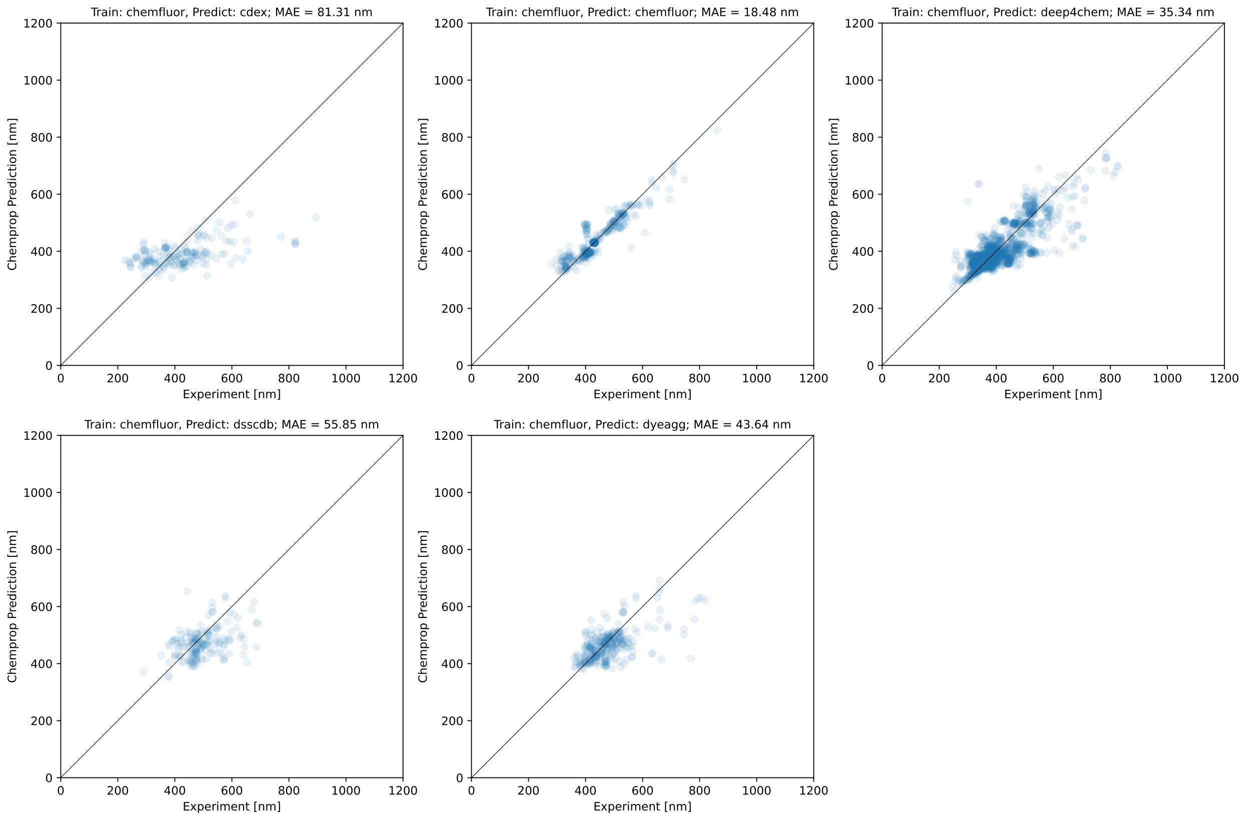

Figure S15: **Predictions on all datasets after training on ChemFluor dataset.**

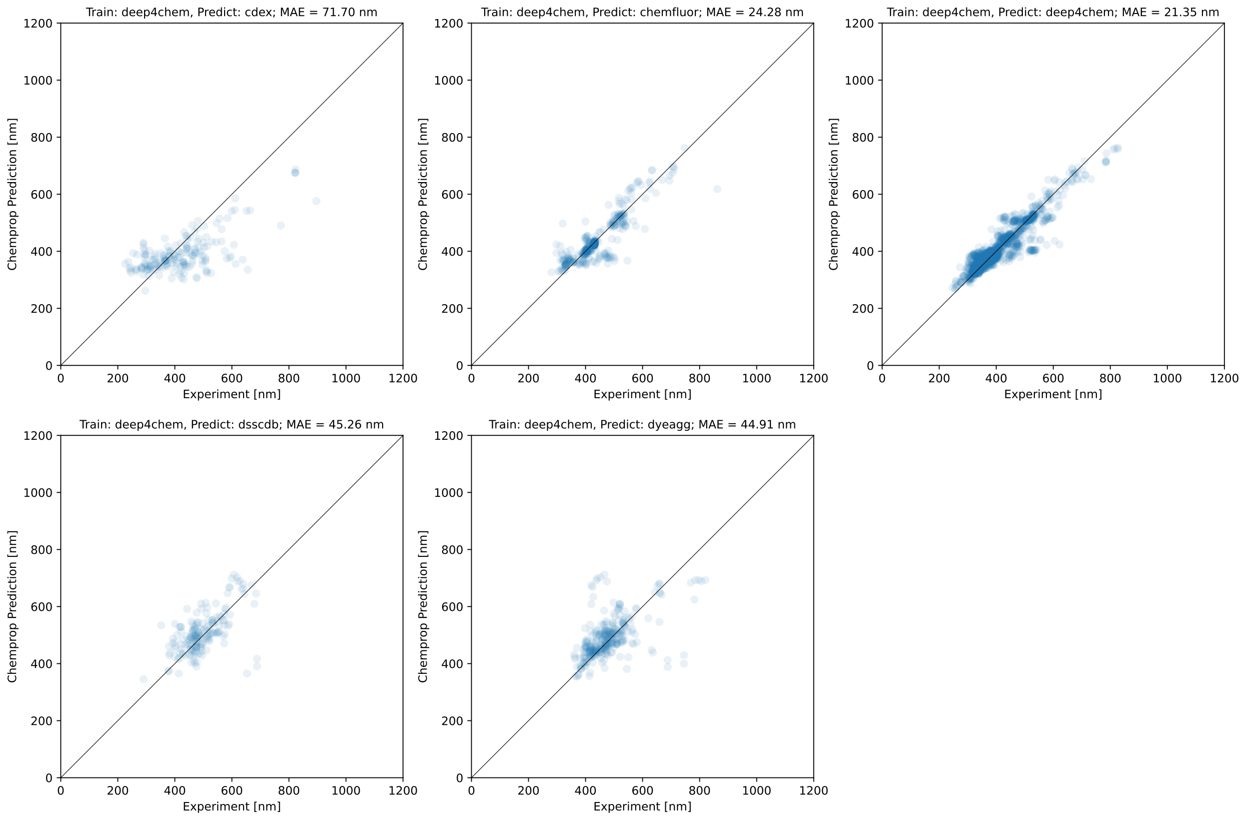

Figure S16: **Predictions on all datasets after training on Deep4Chem dataset.**

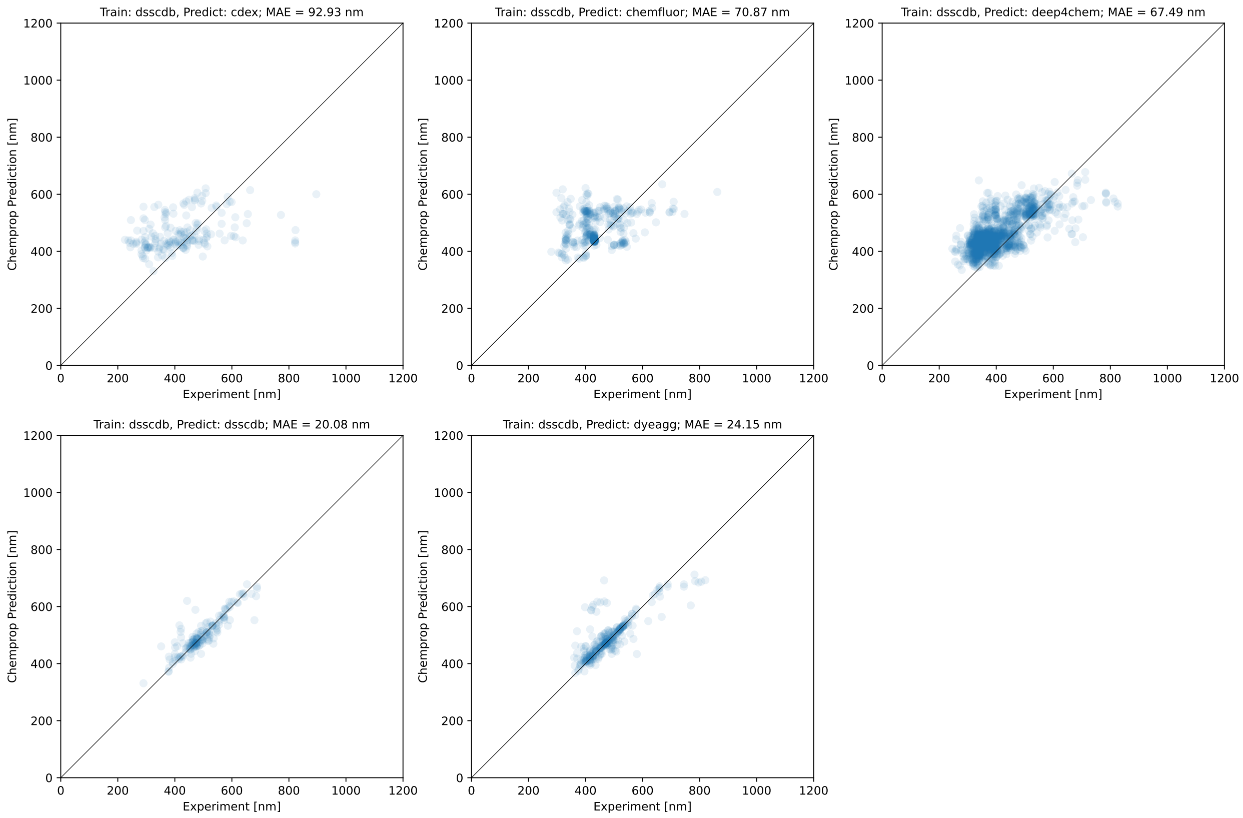

Figure S17: **Predictions on all datasets after training on DSSCDB dataset.**

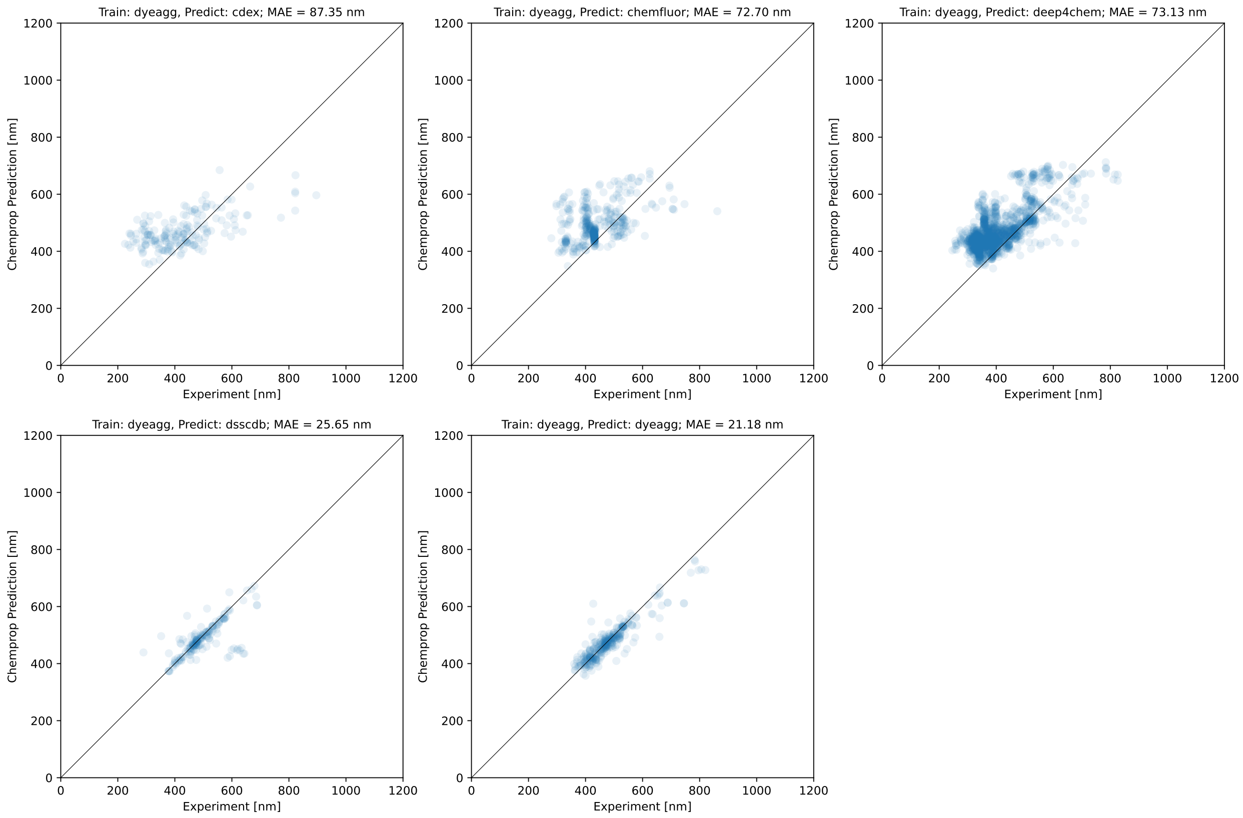

Figure S18: **Predictions on all datasets after training on DyeAgg dataset.**

Transfer Learning Baseline

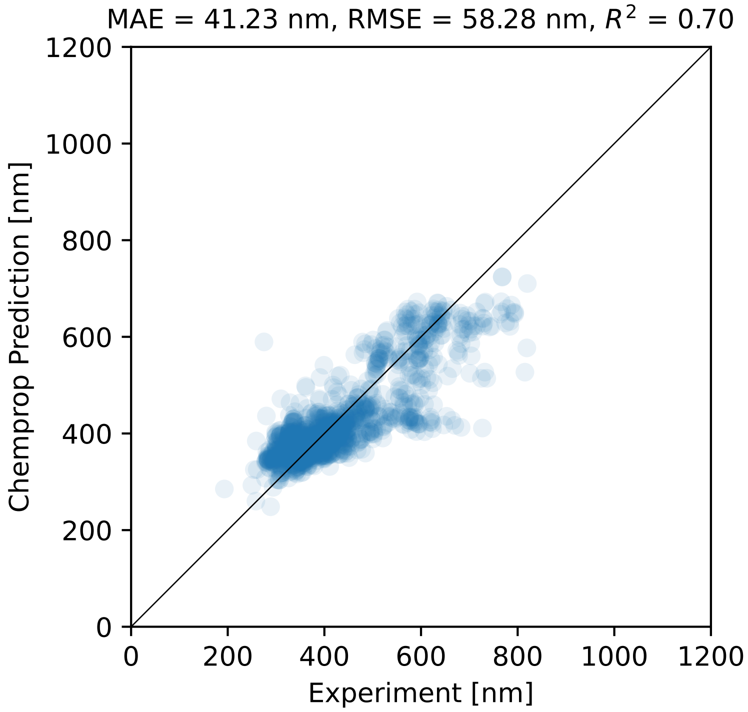

Figure S19: **Predicted Peak Wavelength vs. True Peak Wavelength Using Transfer Learning.** A model was pretrained on TD-DFT data and the D-MPNN parameters were frozen while training on experimental data. CGSD were used to represent the solvent for the experiments. This transfer learning approach performs much worse than our multi-fidelity approach.

UMAP of D-MPNN Embeddings

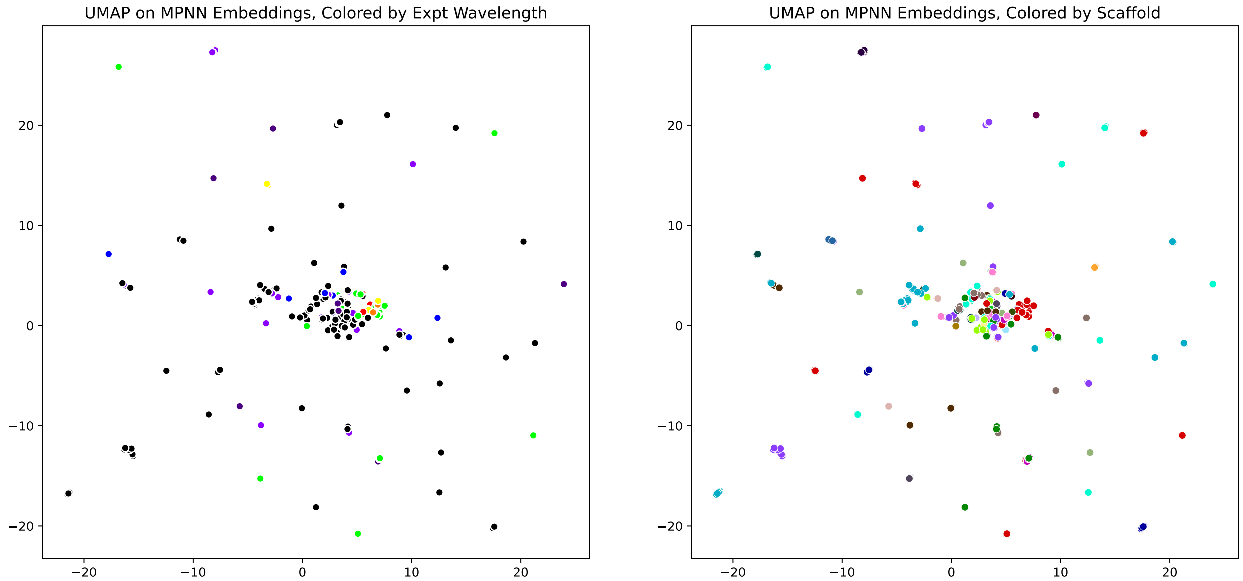

Figure S20: **MPNN Embedding Interpretability through UMAP.** (Left) UMAP plot of molecule D-MPNN embeddings from the scaffold-split test set of the Deep4Chem dataset, using the Chemprop molecule representation and SolventMPNN solvent representation, colored by the experimental peak wavelength of maximum absorption. Colors outside the visible spectrum are shown as black. (Right) Same as (Left), but colored by dye family scaffold.

Analysis of Error in Models

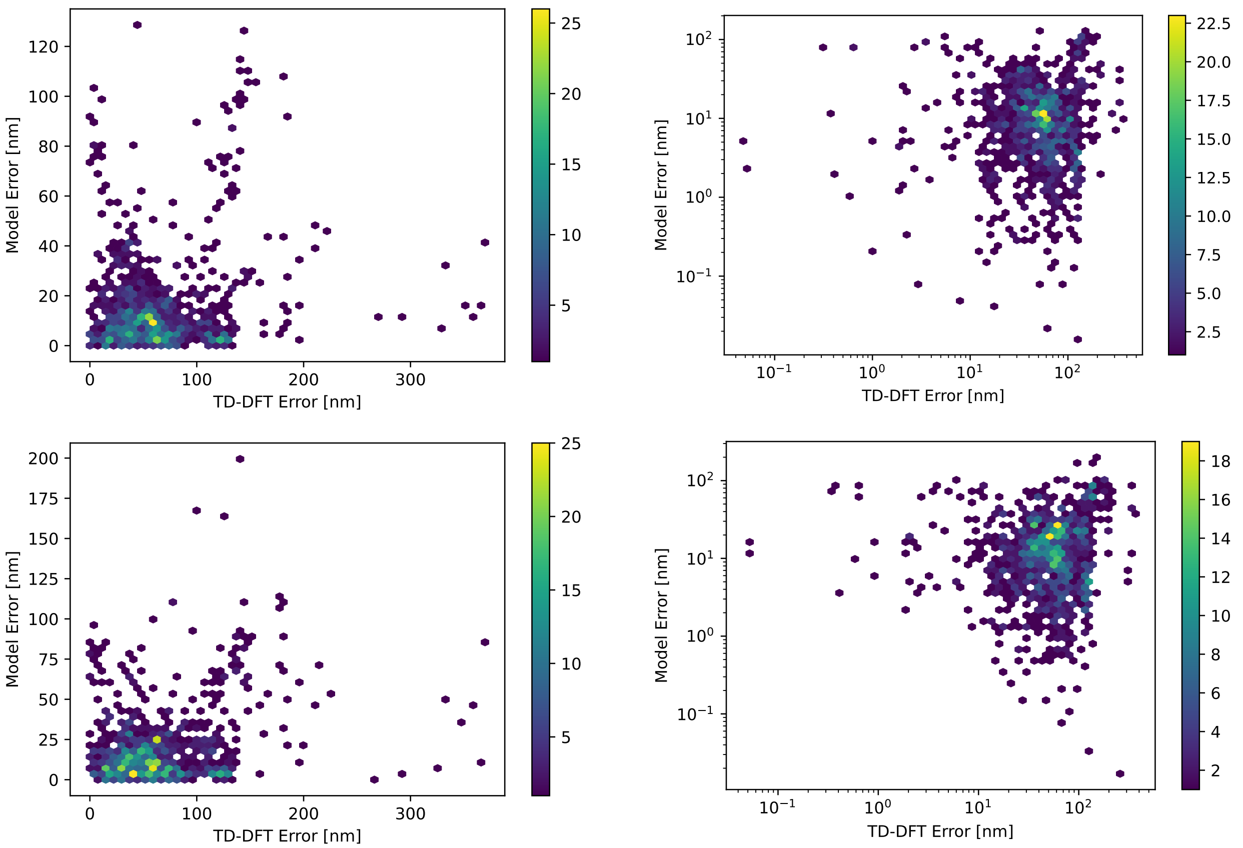

Figure S21: **Error in Models vs. Error in TD-DFT.** (Upper Left) Chemprop model predictions, linear scale; (Upper Right) Chemprop model predictions, log scale; (Lower Left) ChempropMultiFidelity model predictions, linear scale; (Lower Right) ChempropMultiFidelity model predictions, log scale.

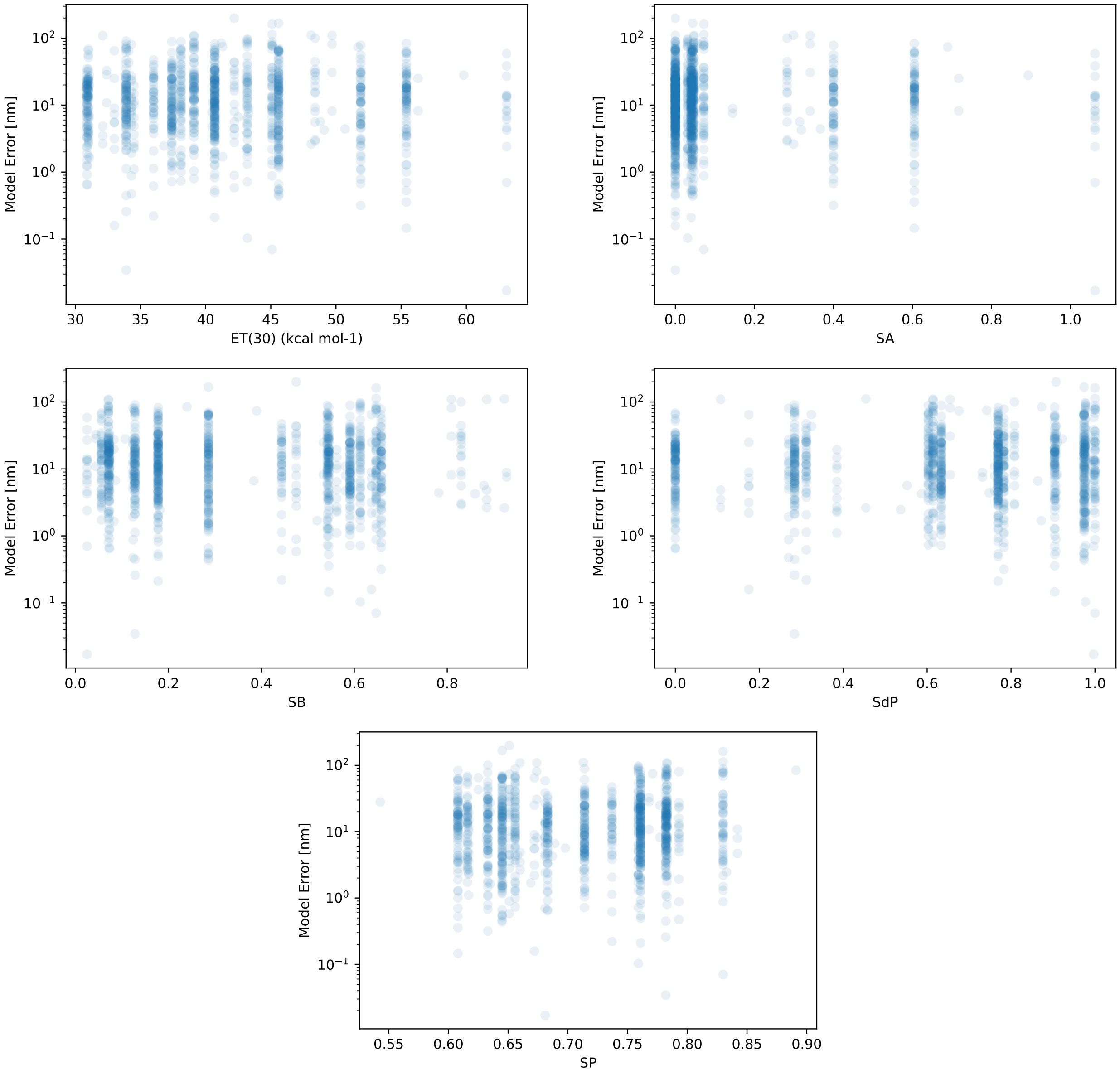

Figure S22: **Error in Models vs. CGSD.**

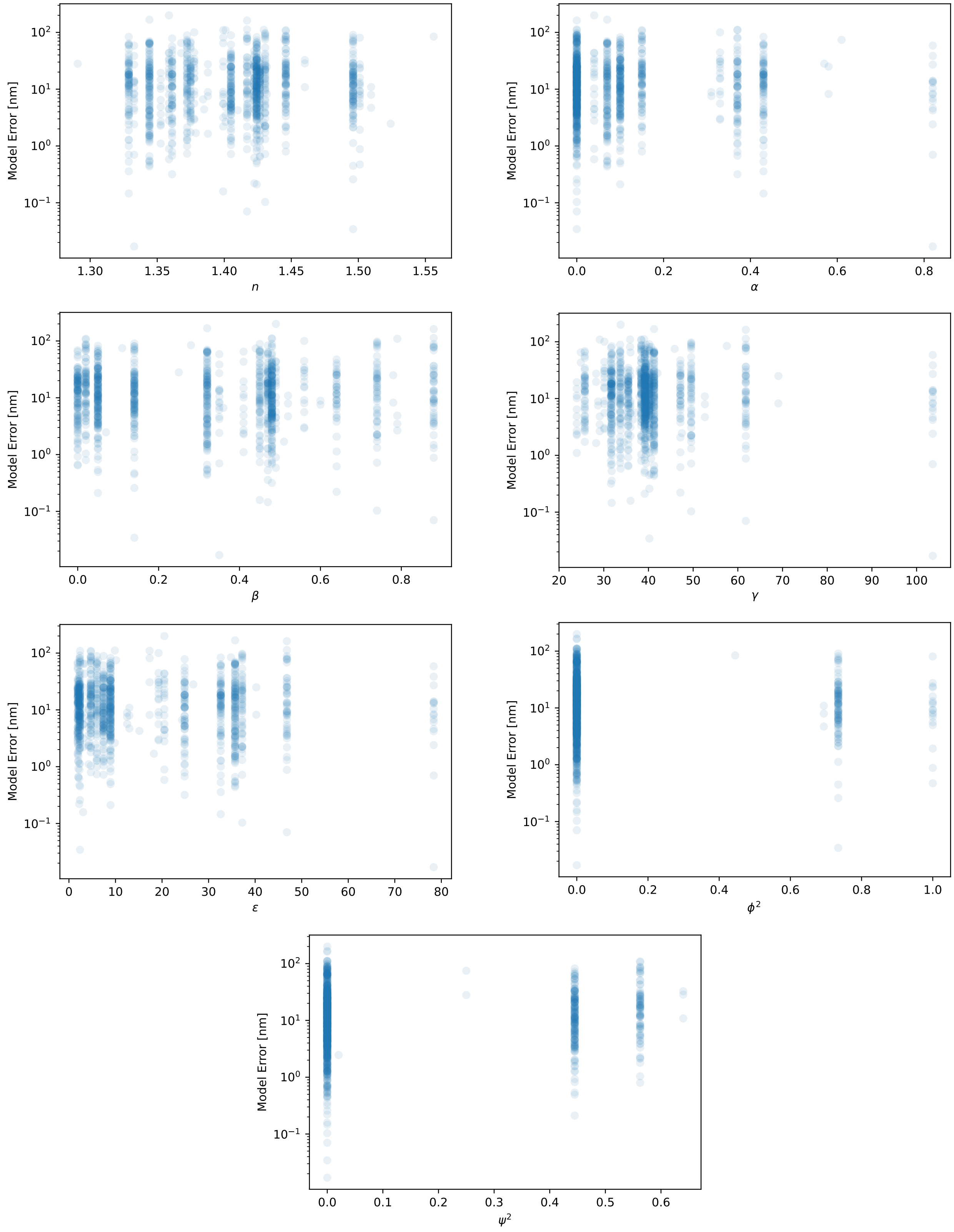

Figure S23: **Error in Models vs. Minnesota Descriptors.**

References

(1) Ju, C. W.; Bai, H.; Li, B.; Liu, R. Machine Learning Enables Highly Accurate Predictions of Photophysical Properties of Organic Fluorescent Materials: Emission Wavelengths and Quantum Yields. *Journal of Chemical Information and Modeling* **2021**, *61*, 1053–1065.

(2) Reichardt, C. Solvatochromic dyes as solvent polarity indicators. *Chemical Reviews* **1994**, *94*, 2319–2358.

(3) Catalán, J. Toward a generalized treatment of the solvent effect based on four empirical scales: Dipolarity (SdP, a new scale), polarizability (SP), acidity (SA), and basicity (SB) of the medium. *Journal of Physical Chemistry B* **2009**, *113*, 5951–5960.
